# Changes of cognitive functions and proinflammatory cytokines across the lifespan in latent *Toxoplasma gondii* infection

**DOI:** 10.1016/j.bbih.2025.101105

**Published:** 2025-09-15

**Authors:** Patrick D. Gajewski, Peter Bröde, Maren Claus, Klaus Golka, Jan G. Hengstler, Jörg Reinders, Carsten Watzl, Edmund Wascher, Stephan Getzmann

**Affiliations:** a Leibniz Research Centre for Working Environment and Human Factors at TU Dortmund *(IfADo)*, Dortmund, Germany; b German Center for Mental Health (DZPG), Partner Site, Marburg Bochum, Germany

**Keywords:** *Toxoplasma gondii*, Cognition, Cytokines, Adult lifespan, Aging, Learning and memory, Executive functions

## Abstract

**Background:**

Recent findings showed serious consequences of latent *T. gondii* infection on the central nervous system, leading to psychiatric, immunological and cognitive impairments. However, little is known about the temporal dynamics of the latent *T. gondii* infection in respect to immunological and cognitive changes across the adult life span. The present study aims at evaluating the course of cognitive changes across the adult life span in relation to latent *T. gondii* infection and the interplay with proinflammatory cytokines leading to chronic inflammation as a potential origin of the cognitive decline in infected adults.

**Methods:**

In a double-blinded cross-sectional design, data of 218 seropositive and 475 seronegative adults aged between 20 and 88 years were compared regarding crucial cognitive domains: processing speed, working memory, immediate and delayed memory, sustained attention, and executive functions. In a subsample of up to 300 participants, concentrations of proinflammatory cytokines IL-6, IL-8, IL-18, and TNF-α were analyzed to evaluate their interaction with *T. gondii,* and to determine whether the cytokines interact in their effects on cognition across the lifespan.

**Results:**

The results showed an interaction between age and *T. gondii* status, with a decline in cognitive performance in infected, relative to non-infected, older individuals, and the reversed pattern in young to middle-aged adults. Specifically, this pattern was evident in working memory, immediate and delayed recall, as well as switching ability. Age was associated with increased levels of proinflammatory cytokines, and reduced concentration of *T. gondii* antibodies. IL-6, IL-8 and TNF-α levels were negatively associated with *T. gondii* antibody level and cognitive performance. Finally, *T. gondii* interacted with IL-6, IL-8 and TNF-α, predicting superior performance in immediate and delayed memory tasks in younger adults with high levels of *T. gondii* IgG antibodies and cytokines, whereas *T. gondii* IgG antibody and cytokine levels played less of a role for these functions in older age.

**Conclusion:**

The findings support a model of dynamically shifting effects of *T. gondii* and proinflammatory cytokines on the central nervous system and cognition with increasing age, suggesting positive effects of *T. gondii* infections in younger adults, and neuroinflammatory effects in older age presumably due to chronic inflammation. Given the high prevalence of latent toxoplasmosis in the general population and the growing population of older adults, these findings are of relevance for public health.

**Trial registration:**

Clinicaltrials.gov NCT05155397.

## Highlights

• *T. gondii* impairs cognitive performance in older adults and enhances in younger adults.
• Age is associated with reduced *T. gondii* and increased cytokine concentrations.
• Proinflammatory cytokines are correlated with *T. gondii* concentration regardless of age.
• Cytokines and *T. gondii* simultaneously moderated memory performance across the lifespan.
• High levels of *T. gondii* and cytokines in young adults predict enhanced memory functions.

## 1 Introduction

One of the most widespread infections that can affect humans and almost all warm-blooded animals is caused by *Toxoplasma gondii* (*T. gondii*), an intracellular protozoan parasite (Tenter et al., 2000). Around one third of the world's population is affected, although there are huge regional differences (Bigna et al., 2020; Dabritz and Conrad, 2010; Tenter et al., 2000).

The parasite reproduces exclusively in the intestinal tract of the cat family (*Felidae*) and contaminates the soil with the oocysts in the excrements. Other animals such as rodents and birds, but also humans become infected by ingesting sporulated oocysts as intermediate hosts via ingestion of *T. gondii* oocysts from contaminated water, soil, fruit, and vegetables, or undercooked meat. After ingesting, oocysts release tachyzoites, which spread through the entire body, and form tissue cysts in muscles and in the central nervous system. The protozoan parasite may nest in the pivotal target organs, mainly the brain after passing the blood-brain barrier, causing a chronic infection affecting the neurotransmitter balance (Carruthers and Suzuki, 2007). Consequently, *T. gondii* can alter the behaviour of infected rodents or birds by reducing the fear or aversion against cats, which enhance the likelihood of the parasite to be transmitted to its definite host. On this way cats become reinfected by consuming issue cysts in infected rodents or birds and the life cycle of *T. gondii* starts all over again (Tenter et al., 2000).

The infection persists with bradyzoites located within the cysts in the brain which build a physical barrier impenetrable for medication (Xiao et al., 2018). The lifelong persistence of *T. gondii* in humans increases the prevalence of seropositivity with age (Boothroyd and Grigg, 2002; Flegr, 2013; Matta et al., 2021; Webster et al., 2013; Xiao et al., 2018, 2022). For example, the prevalence of *T. gondii* reaches 41 % for adults older than 60 years in the USA, which is higher than in all other age groups (Jones, 2001).

The most common form of the disease is a latent (asymptomatic) infection. Asymptomatic means that no symptoms of the infection are manifest and recognizable either for the person itself or for the environment. However, the latent infection may become highly dangerous when the immune system is impaired, e.g., by HIV/AIDS (Golka et al., 2015). Moreover, several neurological and psychiatric diseases like schizophrenia or other psychotic disorders, anxiety disorders, personality changes, higher risk for traffic accidents, or even suicide tendencies were assumed to be associated with latent *T. gondii* infection in humans (Alvarado-Esquivel et al., 2021; Arias et al., 2011; Dickerson et al., 2014; Flegr, 2013; Johnson and Koshy, 2020; Lindová et al., 2011; Sutterland et al., 2015; Webster et al., 2013).

Beside the neuropsychiatric effects of *T. gondii* infection, it is crucial to evaluate whether latent *T. gondii* infection affects specific cognitive functions essential for goal-directed behaviour and independent living. In the last decade, studies increasingly showed deficits in different cognitive domains associated with latent *T. gondii* infection (Beste et al., 2014; Gajewski et al., 2014, 2016; Gale et al., 2015; Guenter et al., 2012; Nimgaonkar et al., 2016). For example, the study by Shirts et al. (2008) analyzed psychomotor speed and switching ability in middle-aged schizophrenic patients measured by the Trail Making Test (TMT), and found a trend for lower performance in *T. gondii* seropositive individuals. Similarly, Guenter et al. (2012) used a cognitive test battery consisting of memory and executive function tasks such as the mentioned above Trail Making Test, Stroop Test (measuring interference processing), word Fluency Test (measuring verbal fluency), Digit Span Test (measuring short-term memory and working memory) and N-back task (measuring working memory and updating) in otherwise healthy young adults to evaluate the association with *T. gondii* seropositivity, but failed to confirm this relationship. However, in our previous study, we reported significant impairments of cognitive performance in otherwise healthy older adults with latent toxoplasmosis, which affected working memory, verbal fluency, learning capacity and delayed recognition (Gajewski et al., 2014). Later, Mendy et al. (2015) as well as Song et al. (2024) found impairments associated with latent toxoplasmosis of immediate and delayed verbal learning, executive functioning, processing speed, sustained attention, and working memory in a large group of older adults from the NHANES study, confirming our previous results. The finding that chronic *T. gondii* infection has its greatest impact on hippocampus (Berenreiterová et al., 2011) may explain the impaired memory functions.

Further studies showed deficits in an auditory distraction paradigm (Beste et al., 2014) as well as impairments of hearing sensitivity in seropositive older adults, and an inverse effect in seropositive younger ones (Getzmann et al., 2024). In line with this, studies with younger adults showed less consistent effects or even improved performance in IgG-seropositive than seronegative individuals (Flegr et al., 2018; Stock et al., 2014, 2017). In summary, there is some evidence that the effects of latent *T. gondii* infection vary with age: There are reports on beneficial effects in sensory and cognitive performance in younger adults, which reverse around the mid-40s and lead to deterioration with increasing age (Colzato et al., 2021; Getzmann et al., 2024).

In a recent meta-analysis, de Haan et al. (2021) examined the association between *T. gondii* and cognitive performance from 13 studies comprising 13,289 healthy individuals and showed a modest but significant association of *T. gondii* seropositivity and four cognitive domains: processing speed, working memory, short-term verbal memory and executive functioning. However, the authors included different age groups in the analysis (mean age was 46.7 years), which may have influenced the reported association between *T. gondii* seropositivity and cognitive performance due to different effects at younger and older ages. It is therefore necessary to assess the association separately for younger and older adults, or to control for the age of the participants.

A common hypothesis for the mechanisms underlying behavioural changes in chronic *T. gondii* infection is neuroinflammation, which may induce behavioural and cognitive deficits (Ingram et al., 2013). *T. gondii* acts as an inflammation amplifier, which is involved in the etiopathogenesis of neuropsychiatric diseases such as schizophrenia, bipolar disorder, major depression, obsessive–compulsive disorder or suicide (Hamdani et al., 2015; Sutterland et al., 2015; Yuan et al., 2019). Beyond the direct effects of chronic *T. gondii* infection on the central nervous system, this infection affects the immune system, resulting in release of inflammatory mediators and cytokines, which play a pivotal adaptive role in controlling infection and limiting parasite growth (Xiao et al., 2022). *T. gondii* infection induces the synthesis and upregulation of proinflammatory cytokines such as interleukin (IL)-6, IL-8 and tumor necrosis factor alpha (TNF-α), biomarkers of chronic inflammation associated with aging, neurodegenerative disorders and cognitive impairment (Carruthers and Suzuki, 2007; Capogna et al., 2023; Fard et al., 2022; Xiao et al., 2022). Also, IL-18 is a potent proinflammatory cytokine involved in defense against infections (Ihim et al., 2022). Elevated levels of IL-18 have been associated with reduced learning and memory performance and neurodegenerative diseases like Alzheimer's, while deficiency can lead to hippocampal issues and neuropsychiatric symptoms (Alboni et al., 2025). These mediators can pass through the blood-brain barrier (or are synthesized in the brain itself) and generate neuroinflammation in the brain that may lead to death of neurons, impairment of synaptic plasticity, changes in neurotransmitter synthesis, release and reuptake (Abdoli et al., 2020).

As mentioned above, the impact of some endogenous factors on human cognition appears to be larger in older than in younger individuals. A reason could be that the decline of brain resources associated with normal aging may interact with inflammatory processes caused by chronic infections, thereby contributing to the age-associated increase in heterogeneity of cognitive performance (MacDonald et al., 2006). This may also apply to the larger effects on performance in older age, as the effects of chronic toxoplasmosis could increase with the length of time since the onset of infection. For this reason, controlling for age is important, as *T. gondii* seems to affect cognitive changes across the adult lifespan.

The present study aimed at investigating the interaction between latent *T. gondii* infection and neuroinflammatory cytokines, and their impact on cognition across adult lifespan. We therefore investigated the development of the most important cognitive abilities in individuals with asymptomatic chronic *T. gondii* infection compared to a non-infected group across the adult lifespan using quantitative neuropsychological tests and examined whether these trajectories were moderated by proinflammatory cytokines, using data collected in two studies with younger and older adults. According to previous findings, we expected lower cognitive performance in older adults with latent *T. gondii* infection and less consistent or even an opposite pattern in younger adults. Additionally, we expected that inflammatory cytokines indicating activation of the immune system moderate this relationship, which help to evaluate the role of immune system in chronic infection.

## 2 Materials and methods

### 2.1 Participants

A total of 728 participants (mean age: *M* = 48.90, *SD* = 16.56 years, age range 20–88 years, 62.0 % female) participated in the study. The data were collected in two studies: the Dortmund Vital Study (DVS; Clinicaltrials.gov NCT05155397), a prospective cohort study on the changes of cognitive functions across the adult lifespan (*n* = 597, mean age: *M* = 44.16, *SD* = 14.30 years, age range 20–70 years, 61.8 % female, see Gajewski et al., 2022 for study protocol), and the Dortmund Aging Study (DAS), a training study with older participants (*n =* 131, *M* = 70.53, *SD* = 4.57, age range 65–88 years, 62.6 % female, see Gajewski et al., 2011, for study details). Both studies were conducted at the Leibniz Research Centre for Working Environment and Human Factors at TU Dortmund (IfADo). The data sets were pooled for analysis.

The participants of both studies were acquired from the general population in the region of the city of Dortmund, Germany. They reported no serious health problems, where “health” was defined in a broad sense and allowed for a history of some diseases such as cardiovascular, immunological, oncological or hormonal problems. Exclusion criteria were current cardiovascular, psychiatric, neurological, motor or oncologic diseases, or hormonal therapy. Typical age-related diseases like hypertension, hypo- and hyperthyroidism and enhanced cholesterol level and the typical medication such as antihypertensives, blood thinners, cholesterol reducers and hormones did not prohibit participation.

All participants were informed about the scope of the studies and gave written informed consent before any study protocol commenced. The studies were approved by the institutional review board and the Institutional Ethics Committee of IfADo in accordance with the declaration of Helsinki.

### 2.2 Determination of *T. gondii* antibody levels

Venous blood of all individuals was sampled in a standardized way at IfADo, and tested for *Toxoplasma gondii* specific IgG antibodies to identify *T. gondii*-negative (T-neg) and -positive (T-pos) participants. IgG antibody levels were measured separately in both studies in different laboratories using different immunoassays and then combined for use in the present study. The analyses of the Dortmund Vital Study were performed in-house using the IgG ELISA (IBL International, Hamburg, Germany) according to the manufacturer's instructions. The ELISA was washed on a hydroFLEX washer and measured on a GENios plate reader system (both TECAN Group Ltd.; Maennedorf, Switzerland). The sensitivity threshold of the *T. gondii* IgG ELISA is 1.04 IU/mL (Siemens Healthcare Diagnostics, 2010). For classification into *T. gondii*-negative and -positive subgroups, participants with IgG-antibody level below 30 IU/mL (*n* = 433 (72.5 %)) were defined as negative, while those participants with IgG-antibody level higher than 35 IU/mL (*n* = 164 (27.5 %)) were defined as positive, according to the manufacturer's instructions (https://ibl-international.com/en_de/toxoplasma-gondii-igg, accessed on January 16, 2024).

The IgG analyses of the Dortmund Aging Study were performed in a local clinical laboratory in Dortmund (MVZ, Dr. Eberhard & Partner Dortmund GbR; https://www.medizin-zentrum-dortmund.de/en/), using an enzyme immunoassay (EIA) Enzygnost® Toxoplasmosis/IgG (Siemens Healthcare Diagnostics, Eschborn, Germany). The EIA was processed on a BEP III system (Siemens Healthcare Diagnostics, Eschborn, Germany). The sensitivity threshold of Enzygnost® Toxoplasmosis/IgG is at least 6 IU/mL but the cut-off for the clinical application is 10 IU/mL. The upper measurement limit is about 2000 IU/mL with the initial sample dilution of 1 + 230. Sensitivity and specificity after retesting in blood donors (n = 260) with initial equivocal results was 100 %, according to the manufacturer (Siemens Healthcare Diagnostics, 2010). IgG concentrations of 35 IU/ml or higher qualified the participants to the T-pos group (54 participants) and non-infected participants (0 IU/mL) were included into the T-neg group (42 participants). Subjects with IgG concentrations between 1 and 35 IU/mL were excluded from the analysis (35 participants).

The result of the immunoassay on *T. gondii* specific IgG antibodies was unknown to the participants and to the experimenters of the study.

### 2.3 Determination of cytokines

Immunological parameters were determined by a standard procedure using flow cytometry as described in Maydych et al. (2018). Briefly, serum was collected from peripheral blood by centrifugation after coagulation. Serum supernatant was harvested and centrifuged at 10,000×*g* to remove platelets and aggregates. Samples were aliquoted and stored at −80 °C. Serum concentrations of proinflammatory cytokines IL-6, IL-8, IL-18 and TNF-α were measured using the respective beads of the LEGENDplex Human Inflammation Panel 1 (Biolegend) according to the manufacturer's instructions.

A total of 200 serum samples were available from the DVS and 130 from the DAS. As the concentrations of the cytokines were positively skewed, log 10-transformation was applied to yield an approximately normal distribution of the data (Iwata et al., 2016; Pereira et al., 2021). The log10-transformed function is unitless.

### 2.4 Cognitive testing

The neuropsychological tests evaluated different aspects of cognitive processing.

1. Immediate and delayed verbal memory was measured using *Verbal Learning and Memory Test* (VLMT; Helmstaedter et al., 2001), a German version of the Auditory-Verbal Learning Test. Several memory subcomponents can be measured by the test. The test material of the VLMT consists of a learning list and an interference list with 15 words each, which are semantically independent of each other. In addition, there is a recognition list with all words of the learning and interference list as well as 20 additional words. First, the subjects are presented with the learning list orally by the experimenter five times (VLMT 1 to VLMT 5). After each presentation, the words should be reproduced. This is followed by a single presentation of the interference list (VLMT I), which should be reproduced as well. Immediately after this, the participants are asked to reproduce the learning list presented at the beginning (VLMT 6). After about 30 min, which are used for the other tests, the learning list the subjects were asked to recall the original list (VLMT 7). Finally, the recall list, consisting of a total of 50 words, was presented orally (VLMT W). Here the task is to recognize the 15 words of the learning list. For the present study, four crucial memory parameters were selected as dependent variables: *learning capacity*, i.e., the total number of words from the learning list that the subject recalled in 5 trials (VLMT Ʃ 1–5), *memory retrieval after interference*, i.e., number of words reproduced from the original list after interference (VLMT 6)*, delayed memory*, i.e., retrieval of words reproduced from the original list after 30 min (VLMT 7), and *word recognition*, i.e., the number of recognized words from the recognition list minus errors (VLMT W-F), indicating correct retrieval.
2. The *Digit Span* (DS; Oswald and Fleischmann, 1999) test measures short and working memory. In the first part of the task, (DS forward), a series of digits with increasing length were orally given by the experimenter. The number of correctly memorized series of digits indicates short-term memory performance. Correct reproduction of two series with the same length indicates the correct response. The maximal performance is nine numbers.

In the second part of the task, digit span backward (DS backward), the presented number sequences must be repeated in reverse order. A maximum of eight consecutive numbers can be repeated. Two successive series with the same length indicate the correct response. This version of the task assesses working memory capacity. The sum of DS forward and backward reflects the general memory capacity and was used as a dependent variable (max. score 28).

3. The *color-word interference test* (Stroop Test; Oswald and Fleischmann, 1999) measures interference processing. The task consists of three subtasks. In the first task (Stroop 1), color words printed in blank ink are to be read as quickly as possible (e.g., “red”, “green”). The second task (Stroop 2) consists of naming color bars. In the third task - the interference condition (Stroop 3) - the subjects are asked to name a series of color words, in which a color word is printed in a color which did not match the name of the color (e.g., “GREEN” was printed in red). Here, word meaning, and print color are incongruent, requiring inhibition of reading the word as an automated response and to name the color instead. The difference between Stroop 3 and Stroop 2 (Stroop 3-2) measuring interference processing as a component of executive functions represents the dependent variable.
4. The *Trail Making Test* (TMT; Reitan, 1992) is a paper-pencil test consisting of two parts. Part A (TMT-A) measures processing speed by asking the participants to connect the numbers 1 through 25 consistently in ascending order. In part B, the letters A to L and the numbers 1 to 13 are to be alternately connected in ascending order. In this dual task, parallel processing of the two different subtasks “numbers” and “letters” is required. Part B (TMT-B) measures switch ability, a component of executive processing.
5. The Digit-Symbol-Test (DST) test measures aspects of focused attention and psychomotor speed (Oswald and Fleischmann, 1999). In this test, the symbols on the test sheet have to be matched to the numbers 1 to 9 within 90 s. The maximum score is 93. The number of correct number-symbol assignments served as dependent variable.

The performance testing system (LPS), consisting of 14 subtests, was developed by Horn (1983) and measures both fluid and crystallized aspects of intelligence.

6. LPS-3 measures logical reasoning. The aim is to indicate the incongruent element in each row of eight logically arranged symbols. The highest score to be achieved is 40. The respondent was given 5 min to complete the test.
7. The LPS-6 is a verbal fluency test measures word fluency and cognitive flexibility. In this test, the participant is asked to identify as many words as possible from the three given initial letters. For each initial letter the participant was given 1 min. The total number of words written down (without repetition, without rule violation) were used as the test score in the evaluation.
8. The LPS-7 requires a mental rotation of letters in the plane – an ability attributed to fluid intelligence. The task consists of crossing out those symbols that are recognized as mirror images. The time limit for this subtest is 2 min. A maximum of 40 recognized symbols can be achieved. The number of correctly crossed out numbers or letters was used as dependent variable for the evaluation.
9. The Cognitive Failures Questionnaire (*CFQ*; Broadbent et al., 1982) was used to assess performance in daily activities. *CFQ* is a scale including 26 questions related to attentional and memory lapses in the daily life.

For details and all tests included in the test battery please refer to Gajewski et al. (2023a).

### 2.5 Data processing and statistical analysis

The number of participants contributing to the analyzed data varied between measures: *n* = 729 provided blood samples for the analysis of the *T. gondii* status, the number of available cognitive data sets varied depending on a test between *n* = 627 and *n* = 691, and data of the proinflammatory cytokines was available from *n =* 330 participants.

The data analysis was conducted in six steps: In the first step, a regression analysis was conducted to evaluate the relationship between *T. gondii* antibody levels in serum and participants’ age. Additionally, the annual risk of infection was assessed (ARI; Cauthen et al., 2002), indicating the risk of an individual without previous infection being infected over one year of life. ARI is calculated using the prevalence and age of the study group by ARI = 1-(1-P)^1/M^, where P is the estimated seroprevalence of the infection in the study group, and M is mean age of the population.

**Fig. 1:**
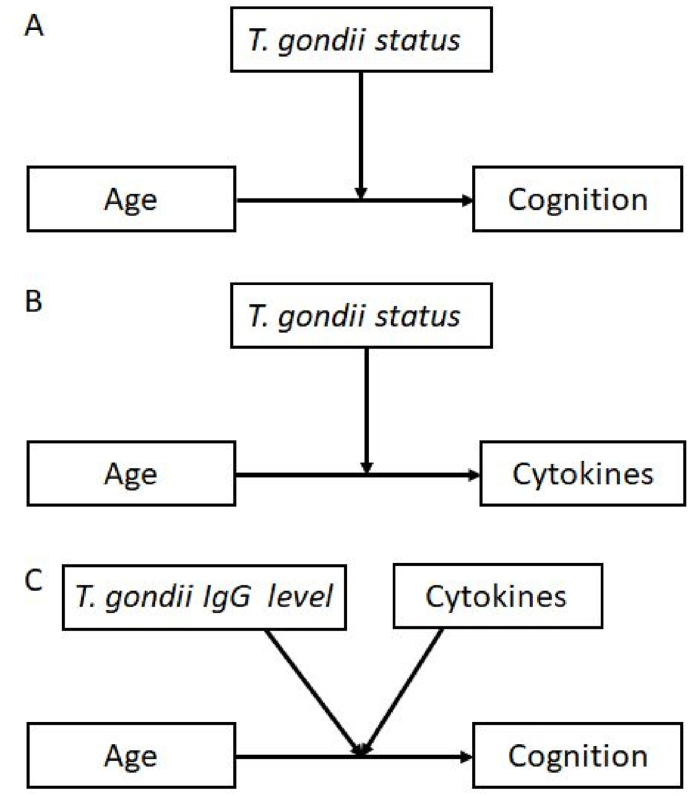
A–C. Regression models used in the study. (A) Moderator analysis with age as independent variable, cognitive function as dependent variable and *T. gondii* status as a moderator variable. (B) Moderator analysis with age as independent variable, proinflammatory cytokine as dependent variable and *T. gondii* status as a moderator variable. (C) Moderator analysis with age as independent variable, cognitive function as dependent variable and two (correlated) moderators *T. gondii* IgG level and cytokines.

In the second step, a series of moderator analyses were conducted using the PROCESS macro for SPSS V. 4.2 (Hayes, 2022), with age as continuous predictor, cognitive test value as continuous dependent variable, and *T. gondii* status as a discrete moderator variable with T-pos and T-neg groups (regression model number 1: Y: cognitive test; X: age; W: *T. gondii,* Fig. 1A). Performance in cognitive tests was plotted against age for T-pos and T-neg participants. Briefly, the Hayes PROCESS macro provides tools for conditional process analysis, which integrates moderation to examine how relationships vary across different contexts. This approach examines how the relationship between an independent variable (X) and a dependent variable (Y) is mediated by a moderator variable (W), and how this mediated relationship is further moderated by another variable (Z). Furthermore, we used the Johnson-Newman technique, which identifies specific values of the moderator variable where the effect of the independent variable on the dependent variable transitions from being statistically significant to non-significant. The Johnson-Newman technique, available within PROCESS, helps determine the regions of significance for the effect of an independent variable on a dependent variable at different values of a moderator. These values indicated substantial differences between the slopes of the regressions for each group. The process macro generates codes for visualizing the interactions.

In the third step, we conducted series of linear regression analyses in a subsample of 330 participants with valid measures of the log10-transformed proinflammatory cytokines (IL-6, IL-8, IL-18 and TNF-α) to test the relationship between the cytokine concentrations and *T-gondii* IgG antibody levels. Following this, in the fourth step, a series of moderator analyses were performed to evaluate whether age predicts the level of the proinflammatory cytokines. In step five we ask, whether this relationship is additionally moderated by *T. gondii* status (T-pos vs. T-neg). Analogically to the second step, the PROCESS macro was used with age as a predictor, cytokine as continuous dependent variable, and *T. gondii* as discrete moderator variable (regression model number 1: Y: cytokine; X: age; W: *T. gondii,* Fig. 1B).

In the final sixth step, we analyzed whether the relationship between age and cognitive functions is moderated simultaneously by *T. gondii* IgG serum antibody level and the proinflammatory cytokines (Fig. 1C) in up to *n* = 296 individuals. We used age as predictor, cognitive tests as continuous dependent variables, log10-transformed *T. gondii* IgG antibody levels of the T-pos individuals and log10-transformed cytokine concentrations (IL-6, IL-8, IL-18, TNF-α) as continuous moderator variables (Y: cognitive test; X: age; W: *T. gondii*; Z: cytokine). As the log10-transformed IgG levels of *T. gondii* antibody levels and the log10-transformed levels of the cytokines were correlated (see section 3.3), model 3 of the PROCESS macro with two dependent moderators was used. The moderator variables were categorized in three groups (high, middle, and low) by the mean (*M*) and one standard deviation below and above the mean (*M* ± 1 *SD*).

The analysis examines potential main effects of age, *T. gondii* IgG levels, and cytokines on cognition, as well as the two-way interactions: *T. gondii* antibody levels x age on cognition, and *T. gondii* antibody levels x cytokine on cognition, and finally, the three-way interaction *T. gondii* antibody levels x age x cytokine on cognition. Δ*R*^*2*^ indicates changes of the *R*^*2*^ by the highest interaction term, i.e., how much more variance the three-way interaction term explains than the moderator and predictor alone. The three models are illustrated in Fig. 1.

## 3 Results

A total of 218 (29.9 %) participants (mean age: *M* = 54.5 years, *SD* = 15.6, age range: 20–88 years, 60.4 % female) were *T. gondii* IgG antibody positive (T-pos, IgG concentration: *M* = 227.2, *SD* = 210.4, IgG range 35–1425 IU/mL). In contrast, 475 (65.2 %) participants (mean age: *M* = 44.7 years, *SD* = 15.3, age range 20–77 years, 63.7 % female) were *T. gondii* IgG antibody negative (T-neg, IgG concentration below 30 IU/mL). One intermediate case with a serum level of 33 UI/mL was assigned to the T-pos group (Getzmann et al., 2024). The Chi-square test (*X*^*2*^ = .58, p = .44) indicates that gender was similarly distributed in T-neg and T-pos groups. Thirty-five (4.8 %) participants (mean age: *M* = 70.4 years, *SD* = 9.2, 50 % female) were excluded from the analysis due to intermediate *T. gondii* IgG antibody levels (1–34 IU/ml). Descriptive statistics of the neuropsychological test scores and levels of the proinflammatory cytokines in serum are included in the Supplementary material (Table S1 and Table S2).

### 3.1 Association between latent *T. gondii* level and age

As the *T. gondii* IgG antibody concentration was not normally distributed (Kolmogorov-Smirnov-*Z* (218) = .072, *p* < .0001), it was log10-transformed for the further analysis. The T-pos subgroup was older than the T-neg subgroup, according to a *t*-test for independent samples (*t* (691) = 9.72, *p* < .001; T-neg: mean age *M* = 44.7 years, *SD* = 15.3; T-pos: mean age *M* = 54.4 years, *SD* = 15.7), probably due to infection rates status accumulating with age.

The prevalence of chronic *T. gondii* infection in the study group was 29.9 % and the mean age of the whole sample was 47.7 years. Thus, the assessed annual rate of new infection per year-of-live (ARI) was 1-(1-.299)^1/47.7^ = .005 (.5 %).

**Fig. 2:**
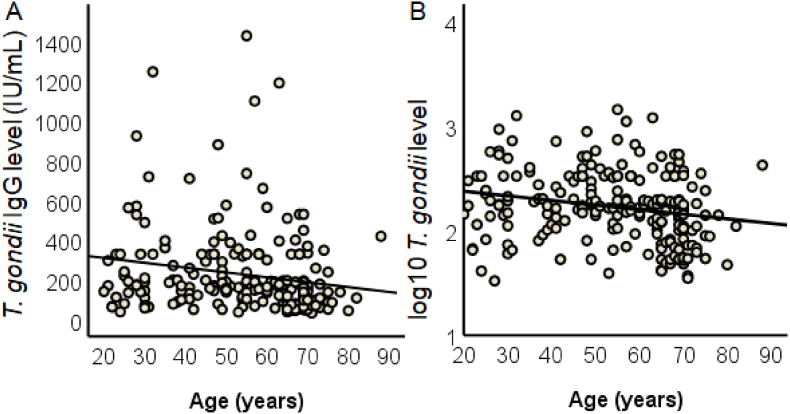
A–B. Individual, absolute (A) and (B) log10-transformed *T. gondii* IgG antibody levels of the 217 seropositive participants with the regression lines.

Fig. 2A shows the distribution of the absolute antibody concentration, and Fig. 2B log10-transformed *T. gondii* IgG antibody concentration in T-pos participants as a function of age with the regression lines. Age negatively predicted the absolute *T. gondii* IgG antibody concentration in the T-pos subgroup (*R*^*2*^ = .03, *F* (1, 217) = 6.64, *p* = .011). Similar results were found for the log10-transformed IgG *T. gondii* levels (*R*^*2*^ = .04, *F* (1, 217) = 9.76, *p* = .002). However, when we test the difference between the two studies (DVS vs. DAS) there is a difference in the mean level of IgG antibodies (t (216) = 3.53, p < .001), which may contribute to the negative association between age and IgG antibody concentrations. This point will be considered in the discussion.

### 3.2 Moderator effects of latent *T. gondii* infection on age-related cognitive changes

**Fig. 3:**
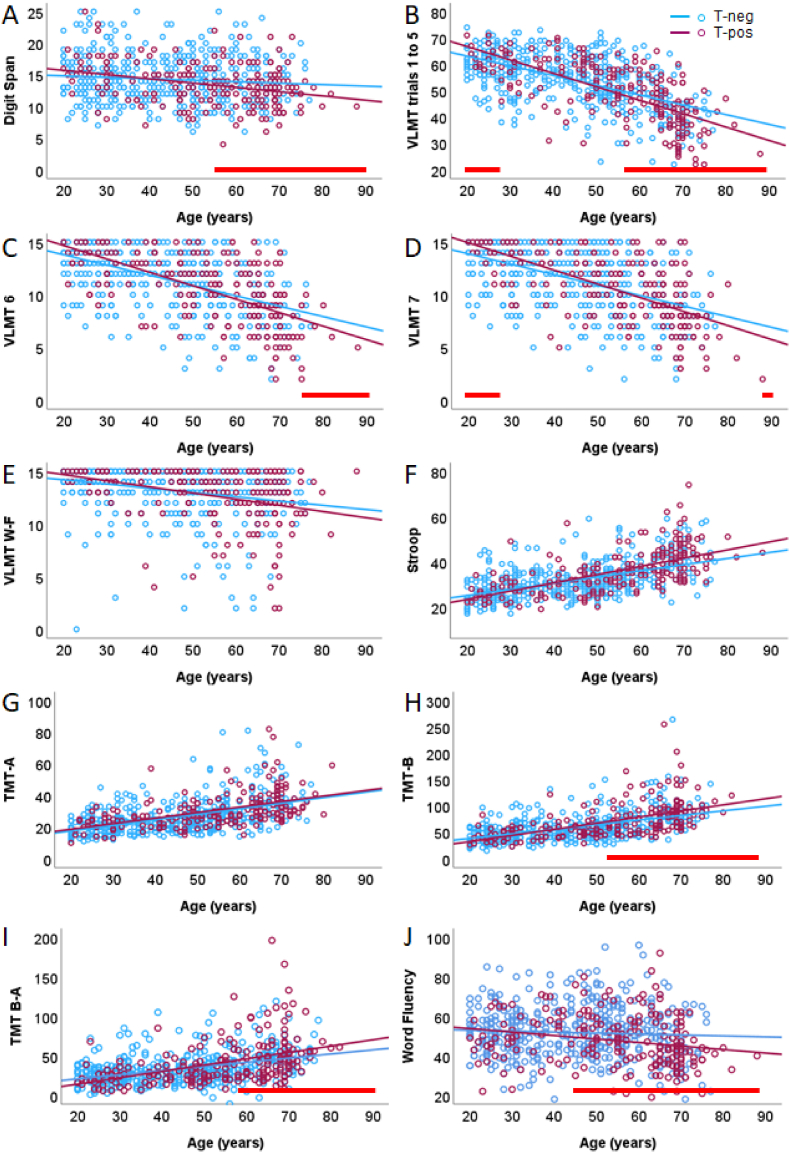
A–J. Cognitive performance as function of age for 218 *T. gondii* IgG seropositive individuals (T-pos, red) and 475 *T. gondii* IgG seronegative participants (T-neg, blue) with regression lines. (A) Digit span Σ forward + backward, (B) VLMT Σ1-5, (C) VLMT-6, (D) VLMT-7, (E) VLMT-W-F, (F) Stroop, (G) TMT-A, (H) (TMT-B), (I) (TMT-B-A), (J) (Word Fluency, WFT). Fig. 3A–E and 3J indicate number of correctly remembered word (i.e., more words indicate better performance). Fig. 3F–I indicate time to complete a task (the shorter, the better). Performance in cognitive tasks declines as a function of age and was partly moderated by *T. gondii* status indicated by significant interaction with age. Johnson-Neyman intervals show the conditional effects of *T. gondii* on cognitive performance as a function of age. Age ranges at which the positive *T. gondii* status had a significant effect on cognitive performance are marked with horizontal red lines. (For interpretation of the references to color in this figure legend, the reader is referred to the Web version of this article.)

The age-related changes of cognitive functions were tested depending on the *T. gondii* status (cf. Fig. 1A). Fig. 3 presents the interactions between age and *T. gondii* status for each of the cognitive functions.

For the digit span backward testing *working memory,* the model was significant (*R*^*2*^ = 5.5 %; *F* (3, 687) = 13.36, *p* < .001). While the effect of *T. gondii* status was not significant (*t* = 1.06, *p* = .108), there was an effect of age on working memory (*t* = −3.03, *p* < .01), indicating a progressive decline of working memory with age. Importantly, *T. gondii* status moderated the regression (Δ*R*^*2*^ = .06 %, *F* (1, 687) = 4.55, *p* < .05) with a larger slope in T-pos than T-neg. The Johnson-Neyman intervals, as illustrated in Fig. 3A, showed that significant differences in predicted digit span test performance emerged from the age of 56 years (*p* = .05) until the age of 88 years (*p* = .012).

Regarding *learning capacity* measured by the sum of trials 1 to 5 of the VLMT (VLMT-Σ1-5), the regression model was significant (*F* (3, 634) = 141.94, *p* < .001), predicting *R*^*2*^ = 40.2 % of the variance. The analysis indicated an effect of *T. gondii* status (*t* = 2.59, *p* < .01) and age (*t* = −13.91, *p* < .001) as well as an interaction of both (Δ*R*^*2*^ = .08 %, *F* (1, 634) = 9.23, *p* < .01). This interaction covered an interesting pattern: The difference between T-pos and T-neg was significant in young adults starting with the age of 20 years (*p* = .026) until the age of 27 years (*p* = .05), and again starting with the age of 57 years (*p* = .050) until the age of 88 years (*p* = .002). As evident from Fig. 3B, performance of the T-pos young adults was higher than of the young T-neg individuals, while the pattern reversed in older adults. The youngest 14.2 % and the oldest 31.8 % of the entire population were thus affected by *T. gondii* status in this task in the opposite way.

The moderation analysis of the *memory retrieval* of learned material after interference (VLMT-6) revealed a significant regression model (*R*^*2*^ = 32.7 %; *F* (3, 631) = 102.13, *p* < .001), with significant effects of *T. gondii* status (*t* = 2.02, *p* < .05), age (*t* = −12.24, *p* < .001), and the interaction of both (Δ*R*^*2*^ = .05 %, *F* (1, 631) = 4.83, *p* < .05). *Memory retrieval* did not significantly differ between the younger T-pos and T-neg (in 20-year-old adults, *p* = .066), but started to differ with the age of 76 years (*p* = .050) until 88 years (*p* = .036) (Fig. 3C).

The regression model for the *delayed memory* retrieval (VLMT-7) was significant (*R*^*2*^ = 32.2 %; *F* (3, 633) = 100.22, *p* < .001). Again, *T. gondii* status (*t* = 2.52, *p* < .05), age (*t* = −12.18, *p* < .001), and the interaction were significant (Δ*R*^*2*^ = .06 %, *F* (1, 633) = 5.15, *p* < .05). The Johnson-Neyman intervals (Fig. 3D) indicated a significant difference in young participants up to 30 years and in the oldest participants aged 88 years and older (*p* < .05). Again, the youngest and the oldest population were affected by *T. gondii* status in the opposite way.

*Delayed recognition* of words (VLMT-W-F) showed a significant regression model (*R*^*2*^ = 8.1 %; (*F* (3, 633) = 18.62, *p* < .001) and an effect of age (*t* = −4.92, *p* < .001), while the effect of *T. gondii* status (*t* = 1.14, *p* = .252) and the interaction with age (*t* = −1.29, *p* = .194) were not significant (Fig. 3E).

The *Stroop interference* (Fig. 3F) measured as the time difference between the interference list and simple color list explained *R*^*2*^ = 31.9 % of the variance (*F* (3, 623) = 97.33, *p* < .001), and showed an effect of age (*t* = 12.43, *p* < .001), but no effect of *T. gondii* status (*t* = −1.30, *p* = .183). The interaction did not reach significance (Δ*R*^*2*^ = .02 %, *F* (1, 623) = 2.10, *p* = .148).

The analysis of the Trail Making Test (TMT-A) assessing *speed of processing* revealed that the regression model explained *R*^*2*^ = 28.0 % of the variance (*F* (3, 625) = 81.00, *p* < .001). There was a main effect of age (*t* = 11.85, *p* < .001), but there was no effect of *T. gondii* (*t* < 1) or interaction of *T. gondii* status and age (Δ*R*^*2*^ = .00 %, *F* (1, 625) < 1, Fig. 3G).

The regression model with Trail Making Test part B (TMT-B) assessing *task switching and cognitive flexibility* explained *R*^*2*^ = 29.9 % of the variance (*F* (3, 625) = 88.70, *p* < .001). While the effect of *T. gondii* status was not significant (*t* = −1.51, *p* = .132), the effect of age (*t* = 11.15, *p* < .001), and the interaction *T. gondii* x age were significant (Δ*R*^*2*^ = .05 %, *F* (1, 625) = 4.17, *p* < .05). The Johnson-Neyman intervals indicated significant T-pos and T-neg differences in participants older than 56 years (*p* = .050) to the oldest ones (*p* = .014, Fig. 3H).

The difference TMT-B and TMT-A explained *R*^*2*^ = 18.7 % of the variance (*F* (3, 625) = 47.76, *p* < .001). The effect of *T. gondii* status was not significant (*t* = −1.87, *p* = .062), while the effect of age (*t* = 7.62, *p* < .001) and the interaction *T. gondii* status x age were significant (Δ*R*^*2*^ = .07 %, *F* (1, 625) = 5.27, *p* < .05). The Johnson-Neyman intervals indicated significant T-pos and T-neg differences in participants older than 59 years (*p* = .050) to the oldest ones (*p* = .012, Fig. 3I).

*Word fluency* (WFT) showed a significant regression model (*R*^*2*^ = 3.7 %; (*F* (3, 633) = 8.06, *p* < .001). There was no effect of age (*t* = −1.15, *p* = .251), and no effect of *T. gondii* status (*t* < 1), but there was a trend for the interaction of *T. gondii* status with age (*t* = −1.82, *p* = .069). The Johnson-Neyman intervals indicated significant T-pos and T-neg differences in participants older than 45 years (*p* = .050) to the oldest ones (*p* = .001, Fig. 3J).

Analyses of the Digit Symbol Test (DST), logical reasoning (LPS-3), Word Fluency Test (LPS-6), mental rotation (LPS-7) or the attentional failures in daily life (CFQ) showed consistent effects of age (all *p*-values <.001), but did not reveal any significant main effect of *T. gondii* status (all *t*-values <1), or interactions *T. gondii* status x age (all *F-value*s < 1).

### 3.3 Association between *T. gondii* IgG level and cytokine levels

To assess the association between the levels of log10 transformed cytokines and log10 transformed *T. gondii* level, a series of linear regression analyses was conducted.

**Fig. 4:**
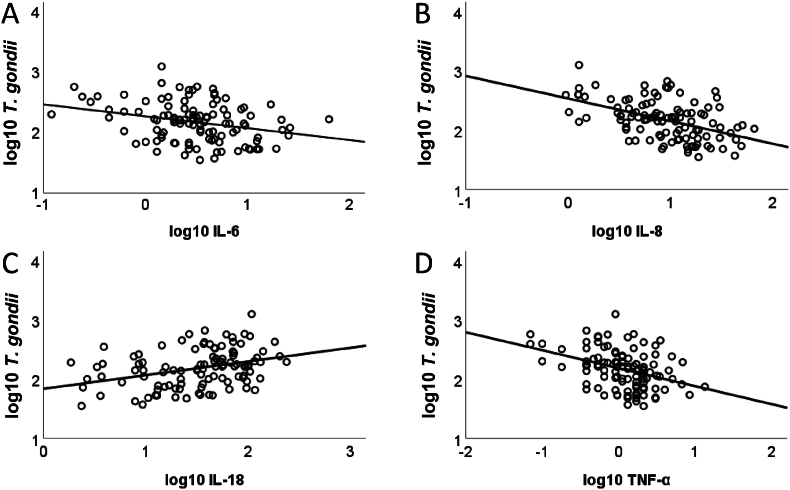
A–D. The log10-transformed *T. gondii* IgG level as a function of log10 transformed concentrations of (A) IL-6, (B) IL-8, (C) IL-18, (D) TNF-α. Concentrations of IL-6, IL-8, and TNF-α decrease with increasing *T. gondii* level. Concentration of IL-18 increases with increasing *T. gondii* IgG level.

As evident from Fig. 4A–T *gondii* antibody level negatively predicted the level of IL-6 (*R*^*2*^ = .09, *F* (1, 110) = 10.5, *p* = .002). A corresponding but more pronounced pattern was found for IL-8 (Fig. 4B, *R*^*2*^ = .23, *F* (1, 108) = 32.2, *p* < .001) and TNF-α (Fig. 4C, *R*^*2*^ = .15, *F* (1, 107) = 18.5, *p* < .001). In contrast, *T. gondii* positively predicted the IL-18 level (Fig. 4D, *R*^*2*^ = .12, *F* (1, 104) = 14.0, *p* < .001).

### 3.4 Moderator effects of latent *T. gondii* infection on age-related changes of cytokines

**Fig. 5:**
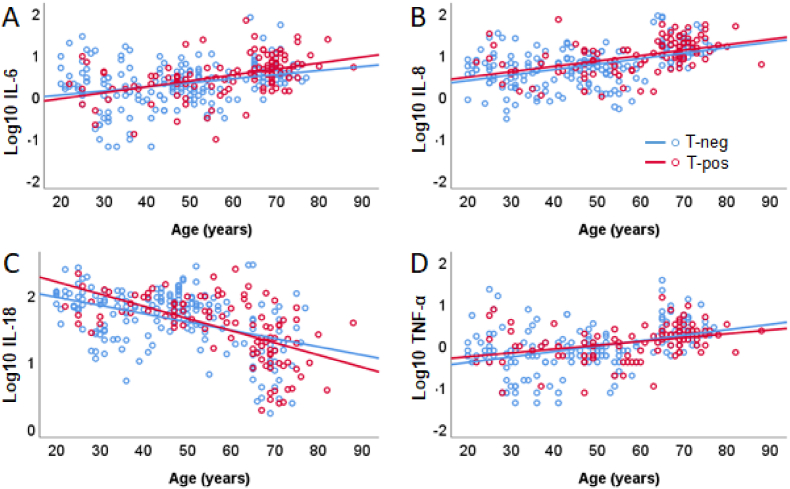
A–D. The log10-transformed proinflammatory cytokines as a function of age for *T. gondii* IgG-seropositive (T-pos, red) and *T. gondii* IgG-seronegative (T-neg, blue) participants with regression lines, shown for (A) IL-6, (B) IL-8, (C) IL-18, (D) TNF-α. Concentration of the cytokines changed as a function of age, but no significant interactions between *T. gondii* status and the cytokines were observed. (For interpretation of the references to color in this figure legend, the reader is referred to the Web version of this article.)

The analysis of log10-transformed concentrations of IL-6 (*n* = 296) as moderator (Fig. 5A) with age and *T. gondii* IgG antibody seropositivity (*n =* 111) vs. seronegativity (*n =* 185) revealed a significant regression model (*F* (3, 292) = 16.98, *p* < .001), predicting *R*^*2*^ = 14.9 % of the variance. The analysis indicated an effect of age, due to an increase of IL-6 with age (*t* = 4.32, *p* < .001), and no effect of *T. gondii* status (*t* < 1). There was no interaction age x *T. gondii* status (Δ*R*^*2*^ = .04 %, *F* (1, 292) = 1.47, *p* = .236). A similar pattern was observed regarding log10-transformed IL-8 (*n* = 293, Fig. 5B) with *n =* 109 T-pos vs. *n =* 184 T-neg participants, showing a significant model (*F* (3, 289) = 31.60, *p* < .001), predicting *R*^*2*^ = 24.7 % of the variance. There was again an effect of age, suggesting an increase of IL-8 level with age (*t* = 7.02, *p* < .001), and no effect of *T. gondii* status (*t* < 1). There was no interaction age x *T. gondii* status (Δ*R*^*2*^ = .0 %, *F* (1, 289) < 1).

The analysis of the log10-transformed IL-18 (*n* = 286) with *n =* 105 T-pos and *n =* 181 T-neg participants yielded a significant model (*F* (3, 282) = 33.84, *p* < .001), predicting *R*^*2*^ = 26.5 % of the variance. There was a reduction of IL-18 with age (*t* = −6.55, *p* < .001), and an effect of *T. gondii* status (*t* = 1.96, *p* = .05) with lower concentrations in *T. gondii* IgG antibody in seropositive than -negative participants. There was a trend for the interaction age x *T. gondii* status (Δ*R*^*2*^ = .09 %, *F* (1, 282) = 3.47, *p* = .063), suggesting lower concentrations in the *T. gondii* IgG antibody seropositive vs. the seronegative group in older age, and the opposite in younger adults (Fig. 5C).

Finally, the analysis of the log10-transformed TNF-α (*n* = 273) with *n =* 108 T-pos and *n* = 165 T-neg individuals (Fig. 5D) showed again a significant model (*F* (3, 269) = 16.03, *p* < .001), predicting *R*^*2*^ = 15.2 % of the variance. There was an increase of TNF-α with age (*t* = 5.07, *p* < .001), but no effect of toxoplasmosis status (*t* = 1.00, *p* = .316), and no interaction of both (Δ*R*^*2*^ = .03 %, *F* (1, 269) = 1.07, *p* = .302).

### 3.5 Correlational analyses for the relationship between cytokines and cognitive functions

As shown in Table 1, there were negative correlations between the log10-transformed IL-6, IL-8 and TNF-α concentrations and memory parameters: VLMT-Σ1-5, VLMT-6, VLMT-7, VLMT-W-F, digit span backward and the word fluency (LPS-6). Positive correlations were found between the log10-transformed IL-6 levels and the time to perform Stroop 2, Stroop 3 and their difference (Stroop 3 – Stroop 2), as well as TMT-A and TMT-B and their difference (TMT-B – TMT-A). This suggests lower memory performance and slower responses in executive control tasks with higher concentrations of the cytokines. The opposite pattern was observed for IL-18, suggesting that higher concentrations are associated with increase in memory performance and faster responses.

**Table 1:** Pearson correlation coefficients for the association between the age, proinflammatory cytokines IL-6, IL-8, IL-18 and TNF-α, and cognitive tests. Two-tailed significance levels are indicated: ∗*p* < .05, ∗∗*p* < .01, *∗∗∗*p < .0001. *Italics* indicates substantial correlations after Bonferroni adjustment (*p* < .05/68 = .00073). The correlations between the cognitive tests and cytokines disappeared after controlling for age.

Test	Age	IL-6 (*n* = 296)	IL-8 (*n* = 293)	IL-18 (*n* = 286)	TNF-α (*n* = 273)
VLMT-Σ1-5	*-,683∗∗∗*	*−.307∗∗∗*	*−.486∗∗∗*	*.487∗∗∗*	*−.356∗∗∗*
VLMT-6	*−.619∗∗∗*	*−.280∗∗∗*	*−.425∗∗∗*	*.401∗∗∗*	*−.295∗∗∗*
VLMT-7	*−.635∗∗∗*	*−.282∗∗∗*	*−.429∗∗∗*	*.394∗∗∗*	*−.314∗∗∗*
VLMT-W-F	*−.311∗∗∗*	−.156∗∗	−.178∗∗	.073	−.127∗
DS-Forward	*−.221∗∗∗*	.032	−.034	.085	.026
DS-Backward	*−.338∗∗∗*	−.114	*−.250∗∗∗*	*.233∗∗∗*	−.135∗
Stroop 2	*.251∗∗∗*	.014	.109	−.170∗∗	.075
Stroop 3	*.608∗∗∗*	*.236∗∗∗*	*.348∗∗∗*	*−.423∗∗∗*	*.305∗∗∗*
Stroop 3–2	*.585∗∗∗*	*.310∗∗∗*	*.369∗∗∗*	*−.422∗∗∗*	*.331∗∗∗*
TMT-A	*.510∗∗∗*	*.231∗∗∗*	*.354∗∗∗*	*−.394∗∗∗*	*.234∗∗∗*
TMT-B	*.541∗∗∗*	*.253∗∗∗*	*.381∗∗∗*	*−.371∗∗∗*	*.227∗∗∗*
TMT B – A	*.440∗∗*	*.208∗∗*	*.312∗∗∗*	*−.285∗∗∗*	*.177∗∗*
DST	*−.645∗∗∗*	*−.220∗∗∗*	*−.318∗∗∗*	*.361∗∗∗*	*−217∗∗∗*
LPS-3	*−.496∗∗∗*	−.072	−.208∗∗	−.167∗	−.018
LPS-6	*−.208∗∗*	−.160∗∗	−.253∗∗∗	.302∗∗∗	−.165∗∗
LPS-7	*−.416∗∗∗*	−.076	−.197∗∗	−.156∗	−.050
CFQ	*−.147∗*	−.051	−.066	.003	−.030

All tests correlated highly consistently with age. Inconsistent or no relationships between cognitive tests the cytokine concentrations were observed for the digit span forward, Stroop task, logical reasoning, spatial rotation, and cognitive failures. However, the correlations between the cognitive tests and the cytokines disappeared after controlling for age.

### 3.6 Simultaneous moderator effects of latent *T. gondii* infection and proinflammatory cytokines on age-related cognitive changes

In order to examine simultaneous moderating effects of *T. gondii* infection and cytokines, only the IgG seropositive *T. gondii* individuals with valid cytokine measures were selected.

The log10-transformed level of *T-gondii* IgG antibodies was correlated with the log10-transformed concentrations of IL-6 (*r* = −.296, *p* < .01), IL-8 (*r* = −.481, *p* < .001), IL-18 (*r* = .346, *p* < .001), and TNF-α (*r* = −.386, *p* < .001). After controlling for age, the correlations were still significant for IL-8 (*r* = −.301, *p* < .001), IL-18 (*r* = .255, *p* < .005), and TNF-α (*r* = −.340, *p* < .001), but no longer for IL-6 (*r* = −.102, *p* = .262).

**Fig. 6:**
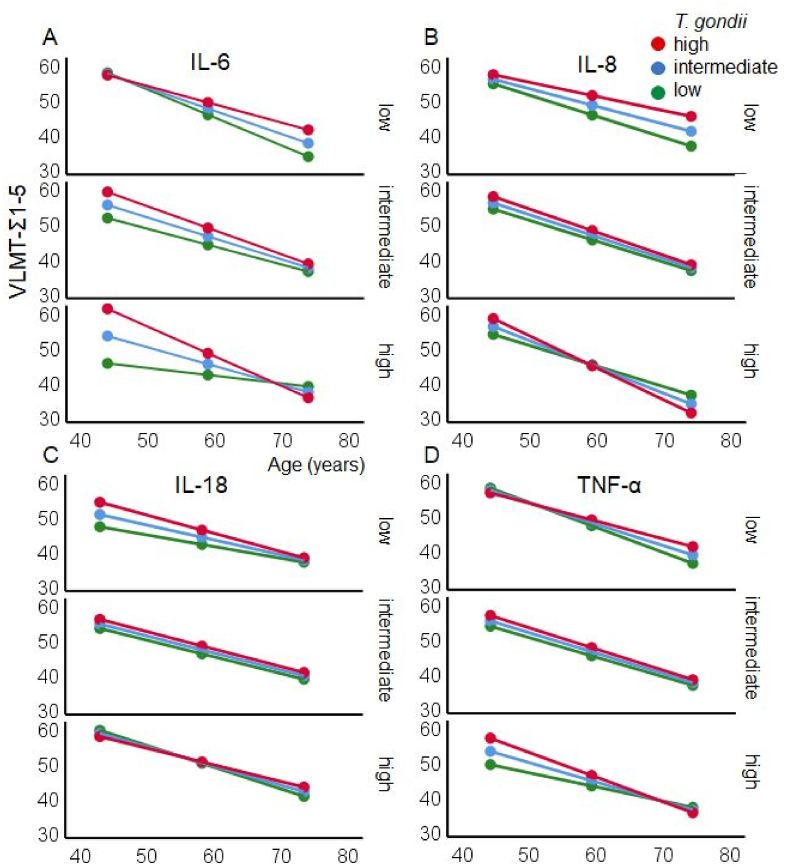
A–D. The linear association between the learning ability (total score of trials 1 to 5 of the VLMT: Y-axis) and age (X-axis) *M* + 1 *SD* (42, 59, 74 years old) was moderated by the log10-transformed *T. gondii* IgG antibody levels (*M* + 1 *SD*: red = high, *M*: blue = intermediate, *M* - 1 *SD* green = low) and the concentration of log10-transformed proinflammatory cytokines IL-6 (A), IL-8 (B), IL-18 (C) and TNF-α (D), (*M* - 1 *SD*: low = upper panel, *M*: intermediate = middle panel, *M* + 1 *SD*: high = lower panel). Please note that in the figure the continuous variables age, *T. gondii* IgG antibody level, and concentration of cytokines were categorized in three groups each, with mean (M) and one standard deviation (SD) below and above the mean (M ± 1 SD). (For interpretation of the references to color in this figure legend, the reader is referred to the Web version of this article.)

The moderation analysis of *learning capacity* (VLMT-Σ1-5) with age as independent variable and IL-6 concentration and *T. gondii* IgG antibody level as moderators explained 61.1 % of the variance in the regression model. Table 2 shows that there was an effect of age and IL-6, but not of *T. gondii* IgG level. Furthermore, there were interactions *T. gondii* IgG level x IL-6, age x IL-6, and a trend for the interaction age x *T. gondii* IgG level. More importantly, however, was the three-way interaction age x *T. gondii* IgG level x IL-6 on learning capacity. As shown in Fig. 6A, the performance in younger adults was more pronounced when the concentrations of *T. gondii* IgG level and IL-6 were high. These differences disappeared in middle-age, and reversed in older age, in which the learning performance was generally reduced, particularly in individuals with higher concentrations of IL-6. A similar pattern was observed with TNF-α (Fig. 6D). In contrast, low concentrations in IL-8 and *T. gondii* IgG level were associated with lower performance on older age (Fig. 6B). No interactions were found with IL-18 (Fig. 6C).

**Table 2:** Results of the moderation analyses with the total score of trials 1 to 5 of the VLMT (VLMT-Σ1-5) as dependent variable, age as independent variable, and log10-transformed *T. gondii* IgG antibody level and log10-transformed IL-6, IL-8, IL-18 and TNF-α concentrations as moderator variables. Δ*R*^*2*^ indicates changes of the *R*^*2*^ by the three-way interaction term. Bold text indicates significance (*p* < .05).

Outcome: VLMT-Σ1-5	*b*a)	*SE*a)	*t*a)	*p*a)
Moderator: **IL-6**				
Intercept	133.269 27.628 4.824 .000	27.628	**4.824**	**.000**
*T. gondii*	−21.704	12.032	−1.804	.074
Age	−1.653	.518	**−3.188**	**.002**
IL-6	−204.032	65.849	**−3.098**	**.003**
*T. gondii* x Age	.457	.233	1.963	.052
*T. gondii* x IL-6	89.457	27.771	**3.221**	**.002**
Age x IL-6	3.266	1.022	**3.196**	**.002**
*T. gondii* x Age x IL-6 b)	−1.445	.441	**−3.278**	**.001**
*N* = 107; *R* = .782; *R*^*2*^ = .611; *F* (7, 99) = 22.23; *p* < .001; Δ*R*^*2*^ = .042 b)				
Moderator: **IL-8**				
Intercept	145.480	50.043	**2.907**	**.005**
*T. gondii*	−34.530	20.570	−1.679	.096
Age	−2.122	.933	**−2.274**	**.025**
IL-8	−86.820	64.554	−1.345	.182
*T. gondii* x Age	.821	.397	**2.067**	**.041**
*T. gondii* x IL-8	46.348	27.525	1.684	.095
Age x IL-8	1.783	1.051	1.697	.093
*T. gondii* x Age x IL-8 b)	−.961	.456	**−2.107**	**.038**
*N* = 105; *R* = .781; *R*^*2*^ = .610; *F* (7, 97) = 65.49; *p* < .001, Δ*R*^*2*^ = .018 b)				
Moderator: **IL-18**				
Intercept	−75.792	159.534	−.475	.636
*T. gondii*	60.084	69.243	.868	.388
Age	1.485	2.326	.638	.525
IL-18	93.122	87.609	1.063	.291
*T. gondii* x Age	−.822	1.015	−.810	.420
*T. gondii* x IL-18	−36.562	38.007	−.962	.339
Age x IL-18	−1.271	1.302	−.976	.332
*T. gondii* x Age x IL-18 b) ∗	.528	.569	.927	.356
*N* = 107; *R* = .782; *R*^*2*^ = .611; *F* (7, 99) = 22.23; *p* < .001, Δ*R*^*2*^ = .004 b)				
Moderator: **TNF-α**				
Intercept	73.968 27.628 4.824 .000	27.279	**2.712**	**.008**
*T. gondii*	3.356	12.145	.276	.783
Age	−.590	.438	−1.348	.181
TNF-α	−133.993	62.443	**−2.146**	**.034**
*T. gondii* x Age	.000	.197	.002	.998
*T. gondii* x TNF-α	58.351	26.251	**2.223**	**.029**
Age x TNF-α	2.105	1.008	**2.089**	**.039**
*T. gondii* x Age x TNF-α b)	−.942	.431	**−2.185**	**.031**

*N* = 103; *R* = .764; *R*^*2*^ = .584; *F*(7, 95) = 20.22; *p* < .001, Δ*R*^*2*^ = .021^*b)*^

a) Regression coefficients (b) with standard errors (SE), t- and p-values.

b) ΔR^2^: change in R^2^ due to the three-way interaction term.

**Fig. 7:**
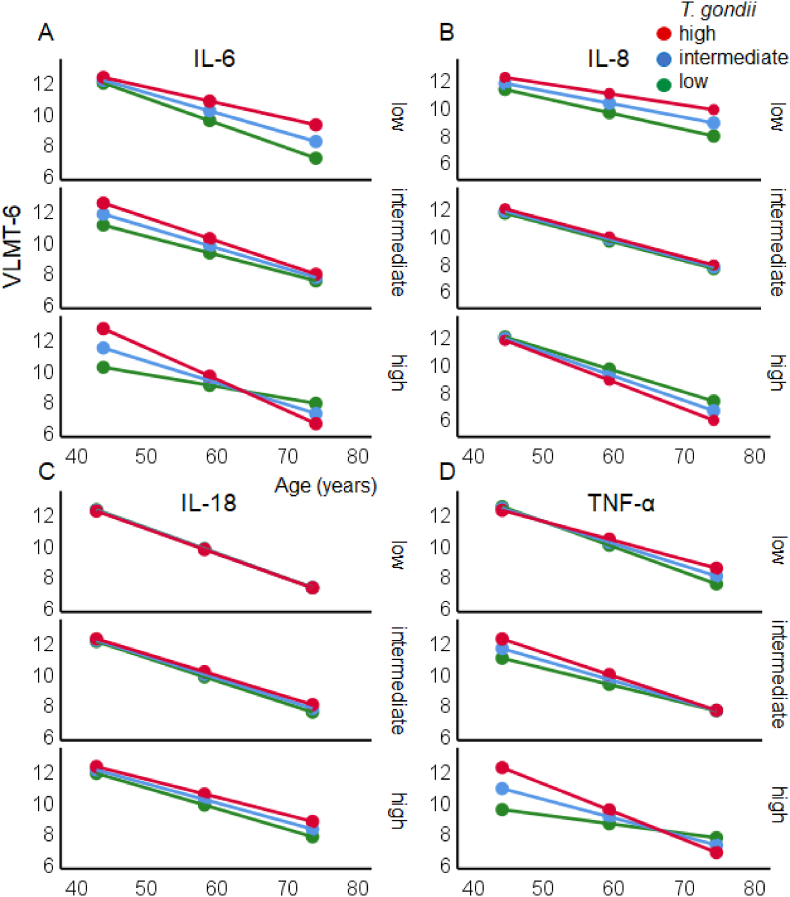
A–D. The linear association between the memory retrieval after interference (total score of the trial 6 of the VLMT: Y-axis) and age (X-axis) *M* + 1 *SD* (42, 59, 74 years old) is moderated by the log10-transformed *T. gondii* IgG antibody level (*M* + 1 *SD*: red = high, *M*: blue = intermediate, *M* - 1 *SD* green = low) and the concentration of log10-transformed proinflammatory cytokines IL-6 (A), IL-8 (B), IL-18 (C) and TNF-α (D), (*M* - 1 *SD*: low = upper panel, *M*: intermediate = middle panel, *M* + 1 *SD*: high = lower panel). Please note that the continuous variables age, *T. gondii* IgG antibody level, and concentration of cytokines were categorized in three groups each, with mean (M) and one standard deviation (SD) below and above the mean (M ± 1 SD). (For interpretation of the references to color in this figure legend, the reader is referred to the Web version of this article.)

Similarly, Table 3 presents the results for VLMT-6 as dependent variable, indicating *memory retrieval* after interference. The regression model with age and IL-6 and *T. gondii* IgG antibody level as moderators explained 45.9 % of the variance and yielded trends for the interactions Age x *T. gondii* IgG antibody level*, T. gondii* IgG level x IL-6, a significant interaction Age x IL-6 as well as an interaction Age x *T. gondii* IgG antibody level x IL-6. As illustrated in Fig. 6, performance in younger adults was increased when the concentrations of *T. gondii* IgG level and IL-6 were high but reduced in older age. Adults with low level of *T. gondii* IgG antibody showed an attenuated pattern (Fig. 7A, low panel).

**Table 3:** Results of the moderator analyses with the total score of trial 6 of the VLMT (VLMT-6) as dependent variable, age as independent variable, and log10-transformed *T. gondii* IgG antibody level and log10-transformed IL-6, IL-8, IL-18 and TNF-α concentrations as moderator variables. Δ*R*^*2*^ indicates changes of the *R*^*2*^ by the three-way interaction term. Bold text indicates significance (*p* < .05).

Outcome: VLMT-6	*b*a)	*SE*a)	*t*a)	*P*a)
Moderator: **IL-6**				
Intercept	26.118	8.794	**2.970**	**.004**
*T. gondii*	−3.693	3.834	−.963	.338
Age	−.342	.165	**−2.066**	**.041**
IL-6	.094	.074	1.271	.207
*T. gondii* x Age	−39.132	21.099	−1.855	.067
*T. gondii* x IL-6	17.616	8.907	**1.978**	**.051**
Age x IL-6	.674	.325	**2.072**	**.041**
*T. gondii* x Age x IL-6 b)	−.310	.140	**−2.211**	**.029**
*N* = 105; *R* = .677; *R*^*2*^ = .459; *F* (7, 97) = 11.75; *p* < .001; Δ*R*^*2*^ = .027 b)				
Moderator: **IL-8**				
Intercept	20.632	15.727	1.312	.193
*T. gondii*	−3.254	6.463	−.504	.616
Age	−.322	.293	−1.099	.274
IL-8	.128	.125	1.023	.309
*T. gondii* x Age	−4.295	20.362	−.211	.833
*T. gondii* x IL-8	4.061	8.685	.468	.641
Age x IL-8	.200	.331	.603	.548
*T. gondii* x Age x IL-8 b)	−.137	.144	−.954	.342
*N* = 103; *R* = .682; *R*^*2*^ = .465; *F* (7, 95) = 11.78; *p* < .001, Δ*R*^*2*^ = .005 b)				
Moderator: **IL-18**				
Intercept	21.235	50.927	.417	.678
*T. gondii*	−.148	22.093	−.007	.995
Age	−.158	.742	−.213	.832
IL-18	−.020	.324	−.060	.952
*T. gondii* x Age	−1.445	27.974	−.052	.959
*T. gondii* x IL-18	−.177	12.129	−.015	.988
Age x IL-18	−.013	.416	−.031	.975
*T. gondii* x Age x IL-18 b) ∗	.024	.182	.130	.897
*N* = 98; *R* = .681; *R*^*2*^ = .464; *F* (7, 90) = 11.14; *p* < .001, Δ*R*^*2*^ = .000 b)				
Moderator: **TNF-α**				
Intercept	10.770	8.401	1.282	.203
*T. gondii*	3.129	3.742	.836	.405
Age	−.052	.135	−.387	.699
TNF-α	−.038	.061	−.621	.536
*T. gondii* x Age	−43.621	19.202	**−2.272**	**.025**
*T. gondii* x TNF-α	18.746	8.072	**2.322**	**.022**
Age x TNF-α	.679	.310	**2.192**	**.031**
*T. gondii* x Age x TNF-α b)	−.301	.132	**−2.269**	**.026**

*N* = 101; *R* = .678; *R*^*2*^ = .460; *F*(7, 93) = 11.32; *p* < .001, Δ*R*^*2*^ = .030 ^*b)*^

a) Regression coefficients (b) with standard errors (SE), t- and p-values.

b) ΔR^2^: change in R^2^ due to the three-way interaction term.

Again, a similar pattern was observed with TNF-α, indicating that memory retrieval was superior in young individuals with a simultaneous high level of *T. gondii* IgG level and TNF-α concentrations. This difference disappeared in the middle-age and reversed in older age, in which the performance was generally reduced (Fig. 7D). No interactions were found with IL-8 and IL-18 (Fig. 7B and C).

**Fig. 8:**
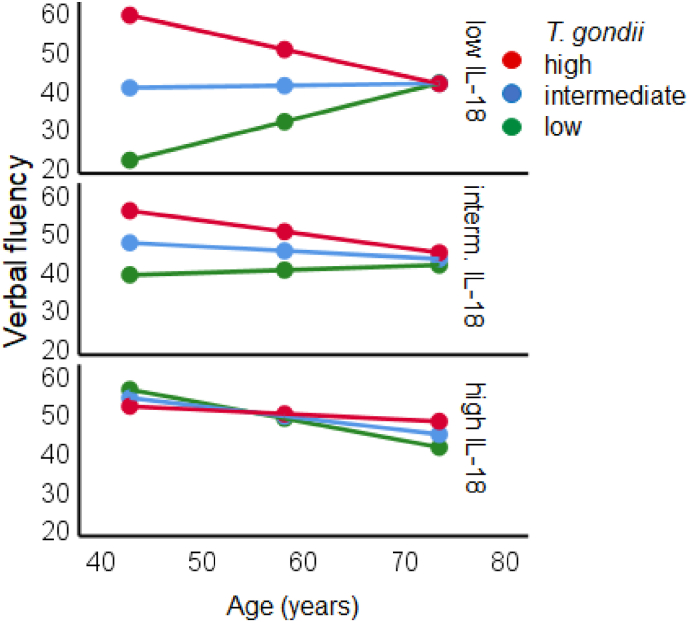
Linear association between verbal fluency (LPS-6: Y-axis) and age (X-axis) *M* + 1 *SD* (42, 59, 74 years old) was moderated by the log10-transformed *T. gondii* IgG antibody level (*M* + 1 *SD*: red = high, *M*: blue = intermediate, *M* - 1 *SD* green = low) and the log10-transformed concentration of IL-18 (*M* - 1 *SD*: low – upper panel, *M*: intermediate = middle panel, *M* + 1 *SD*: high = lower panel). Please note that the continuous variables age, *T. gondii* IgG antibody level, and concentration of IL-18 were categorized in three groups each, with mean (M) and one standard deviation (SD) below and above the mean (M ± 1 SD). (For interpretation of the references to color in this figure legend, the reader is referred to the Web version of this article.)

The moderation analysis of the *verbal fluency* (LPS-6), summarized in Table 4 with age as independent variable, and IL-18 concentration and *T. gondii* IgG antibody level as moderators, explained 24.6 % of the variance in the regression model. Table 4 shows that there was an effect of age, IL-18, and *T. gondii* IgG antibody level. Moreover, there were significant interactions *T. gondii* IgG antibody level x IL-18, age x IL-18, and an interaction age x *T. gondii* IgG antibody level. Notably, however, was the three-way interaction age x *T. gondii* IgG antibody level x IL-18 on verbal fluency. As illustrated in Fig. 8, the performance in younger adults was superior when the *T. gondii* IgG level was high and the level of IL-18 low. In contrast, the performance was reduced with low *T. gondii* IgG level. These differences were attenuated in middle-age and disappeared in older age. Intermediate and high levels of IL-18 diminished this pattern. No interactions were found with IL-6, IL-8 or TNF-α (Table 4).

**Table 4:** Results of the moderator analyses with the verbal fluency (LPS-6) as dependent variable, age as independent variable, and *T. gondii* and log10 transformed IL-6, IL-8, IL-18 and TNF-α concentrations as moderator variables. Δ*R*^*2*^ indicates changes of the *R*^*2*^ by the three-way interaction term. Bold text indicates significance.

Outcome: LPS-5	*b*a)	*SE*a)	*t*a)	*p*a)
Moderator: **IL-6**				
Intercept	4.321	44.034	.098	.922
*T. gondii*	23.344	19.179	1.217	.226
Age	.781	.827	.945	.347
IL-6	−.426	.371	−1.149	.253
*T. gondii* x Age	170.366	105.296	1.618	.109
*T. gondii* x IL-6	−66.797	44.385	−1.505	.136
Age x IL-6	−3.003	1.630	−1.843	.068
*T. gondii* x Age x IL-6 b)	1.199	.703	1.706	.091
*N* = 106; *R* = .431; *R*^*2*^ = .186; *F* (7, 98) = 3.18; *p* < .005; Δ*R*^*2*^ = .024 b)				
Moderator: **IL-8**				
Intercept	24.199	80.011	.302	.763
*T. gondii*	6.737	32.885	.205	.838
Age	.260	1.492	.174	.862
IL-8	−.015	.635	−.023	.981
*T. gondii* x Age	8.017	103.342	.078	.938
*T. gondii* x IL-8	8.612	44.053	.195	.845
Age x IL-8	−.103	1.680	−.061	.951
*T. gondii* x Age x IL-8 b)	−.205	.729	−.281	.779
*N* = 104; *R* = .442; *R*^*2*^ = .196; *F* (7, 96) = 3.38; *p* < .005; Δ*R*^*2*^ = .001 b)				
Moderator: **IL-18**				
Intercept	−675.987	248.274	**−2.723**	**.008**
*T. gondii*	314.154	107.704	**2.917**	**.004**
Age	10.061	3.618	**2.781**	**.007**
IL-18	−4.432	1.577	**−2.810**	**.006**
*T. gondii* x Age	407.350	136.377	**2.987**	**.004**
*T. gondii* x IL-18	−173.476	59.132	**−2.934**	**.004**
Age x IL-18	−5.830	2.027	**−2.876**	**.005**
*T. gondii* x Age x IL-18 b)	2.511	.885	**2.837**	**.006**
*N* = 99; *R* = .496; *R*^*2*^ = .246; *F* (7, 91) = 4.23; *p* < .001, **Δ*R*^*2*^** = **.067**b)				
Moderator: **TNF-α**				
Intercept	63.506	40.914	1.552	.124
*T. gondii*	−.327	18.214	−.018	.986
Age	−.323	.656	−.493	.623
TNF-α	.018	.295	.062	.951
*T. gondii* x Age	123.370	93.569	1.318	.191
*T. gondii* x TNF-α	−53.129	39.332	−1.351	.180
Age x TNF-α	−2.357	1.509	−1.562	.122
*T. gondii* x Age x TNF-α b)	1.028	.646	1.592	.115

*N* = 102; *R* = .406; *R*^*2*^ = .165; *F*(7, 94) = 3.38; *p* < .05; Δ*R*^*2*^ = .023 ^*b)*^

a) Regression coefficients (b) with standard errors (SE), t- and p-values.

b) ΔR^2^: change in R^2^ due to the three-way interaction term.

Apart from an interaction with TNF-α concentration and VLMT-7 as parameter assessing delayed memory performance (Table S1 in supplementary material), no significant interactions with the proinflammatory cytokines and the *T. gondii* IgG antibody level were found for the remaining dependent variables: digit span forward and backward, VLMT-W-F, Stroop 2, Stroop 3, and TMT-A and TMT-B, DST, LPS-3, LPS-7 and verbal fluency (Tables S3–S13 in Supplementary material).

## 4 Discussion

The present study aimed at evaluating the development of cognitive functions in individuals with latent *T. gondii* infection compared to a non-infected group across the adult lifespan. The second aim was to analyze, how these changes were moderated by proinflammatory cytokines. This study design allowed to generate knowledge about the complex interaction between latent *T. gondii* infection and proinflammatory cytokines and the age-related impact on cognition.

First, concurring with previous findings, we observed a decline in cognitive performance with increasing age (Raz and Rodrigue, 2006; Salthouse, 2010). More importantly, *T. gondii* IgG seropositive older adults showed a stronger decline in working memory performance, learning capacity, delayed recall after interference, verbal fluency, and cognitive flexibility compared to the IgG seronegative group, corroborating previous findings (de Haan et al., 2021; Song et al., 2024). This finding suggests accelerated cognitive decline across the lifespan in individuals with chronic toxoplasmosis. Additionally, an intriguing observation was that the *T. gondii* IgG seropositive young adults showed an opposite pattern in some of the parameters, that is, superior cognitive performance in learning capacity and delayed recall than their seronegative peers. This was a somewhat unexpected result, though there was some previous evidence of enhanced cognitive or sensory performance in young adults with latent toxoplasmosis (Stock et al., 2014, 2017; Getzmann et al., 2024). These observations are in line with the framework proposed by Colzato and colleagues (2021), postulating two interrelated mechanisms responsible for the cognitive changes across the lifespan: an increase in catecholamine synthesis, leading to increase of dopamine release (as direct effects of early, latent toxoplasmosis infection), and chronic inflammation, leading to impaired dopaminergic neurotransmission and neurodegeneration (as indirect effects of chronic infection, see Colzato et al., 2021 for details).

The effect of a recent *T. gondii* infection may be the reason for the superior cognitive performance in infected younger adults. In contrast, at older age the time of persistent infection is apparently longer, often lasting for decades, and the effects of chronic inflammation and cumulative, progressive neurodegeneration coincide with the normal age-related decline (Fard et al., 2022; Parlog et al., 2015), leading to impaired cognitive performance compared to non-infected older individuals.

One additional observation should be considered: as evident from Fig. 2, the *T. gondii* IgG antibody concentration decreases with increasing age (but see an alternative account in the section “Limitations”). This is a phenomenon which has also be observed with other antibodies against infectious agents, and is most likely a result of immunosenescence (Pangrazzi and Meryk, 2024). The influence of immunosenescence and the so-called “inflammaging” (see Fard et al., 2022 for review) was also supported by the negative association between *T. gondii* IgG antibody level with proinflammatory cytokines. It could also mean that in older age, due to compromised immune functions, the control of *T. gondii* is impaired which may further explain the observed negative effects of the infection.

The second aim of the study was to elucidate the role of proinflammatory cytokines, indicating activation of the immune system, for the relationship between latent toxoplasmosis und cognitive functions. First, we observed age-related changes of Il-6, IL-8, IL-18, and TNF-α concentrations, corroborating previous reports (Albani et al., 2009; Tylutka et al., 2024). Second, we found consistent correlations between concentrations of proinflammatory cytokines on the one hand, and concentrations of *T. gondii* IgG antibodies on the other. The correlations remained significant after controlling for age. We also found negative relationships between IL-6, IL-8, and TNF-α concentrations and different memory parameters (learning capacity, delayed recall, working memory, verbal fluency) as well as executive functions (interference processing, task switching), confirming previous results (Fard et al., 2022; Singhal et al., 2014, for reviews). However, these relationships were mainly due to the age-factor and disappeared after controlling for age. Therefore, age of the participants should be controlled for, as it is often confounded with age-related changes of biological parameters such as proinflammatory cytokines.

Negative effects of proinflammatory cytokines on memory functions may be mainly due to reduction in hippocampal volumes (Baune et al., 2012). In contrast, IL-18 playing a role in host defence and immune system regulation in inflammatory diseases, showed an opposite pattern, with decreasing concentrations with increasing age and positive association with memory and executive functions regardless of age. However, the functional properties of IL-18 and the role in cognition are understudied yet (Alboni et al., 2025). More important in the present context was the analysis of the interaction between latent toxoplasmosis and the cytokines, and their common impact on cognition across the adult life span. In other words, we asked whether age-related cognitive decline was jointly moderated by proinflammatory cytokines, probably induced by the latent *T. gondii* infection. The results showed significant interactions between latent toxoplasmosis and IL-6, latent toxoplasmosis and IL-8, and latent toxoplasmosis and TNF-α, having a significant impact on the age-related changes in learning capacity and delayed recall measured via different subtests of the Verbal Learning and Memory Tests. More specifically, the result pattern indicated that high *T. gondii* IgG antibody levels and high concentrations of IL-6, IL-8 and TNF-α (but not IL-18) were associated with enhanced word learning and/or immediate recall performance (VLMT-Σ1-5) in young adults, while low *T. gondii* IgG antibody levels and high concentrations of IL-6, IL-8 and TNF-α were associated with lower performance (cf. Fig. 5A, B and 5D). In older age, the role of the *T. gondii* antibodies as well as IL-6 and TNF-α concentrations for the memory performance was diminished. A very similar pattern was found for delayed recall after interference (VLMT-6) for IL-6 and TNF-α (but not for IL-8 and IL-18, Fig. 6). Moreover, it is known that verbal fluency improves with age. In this case, only IL-18, but no other cytokines interacted significantly with this pattern: when IL-18 is low, a low level of *T. gondii* leads to such a typical improvement across age, but when *T. gondii* level is high, this benefit disappears and becomes a disadvantage, meaning that verbal fluency worsens under these circumstances. Other cognitive functions such as working memory, speed of processing, or executive functions were not affected by the interplay between the *T. gondii* IgG antibody level and the concentration of proinflammatory cytokines.

It will be important to identify and understand the underlying mechanisms leading to the memory decline at older age in the context of proinflammatory cytokines, and to a beneficial effect of high *T. gondii* IgG antibody level and high levels of IL-6 or TNF-α at younger age. On the one hand, acute high antibody and cytokine levels are a sign for a strong immune response and therefore a well-functioning immune system. Individuals with a strong immune system may also have better cognitive functions, as suggested by correlations between lower immune age (IMMAX; Bröde et al., 2023) and performance factors such as physical activity (Bröde et al., 2022) or work ability (Gajewski et al., 2023b). On the other hand, chronic inflammation that even increases with age (as indicated by the increase of IL-6, IL-8 and TNF-α concentrations with age in the present study) may lead to neurodegeneration and cause specific functional decrements in brain regions such as the hippocampus and frontal cortex, which are responsible for memory functions (Marsland et al., 2008; Wever et al., 2002), and play a role in stress-related diseases such as burnout (Gajewski et al., 2024). Latent infection with *T. gondii* affects signalling pathways in the brain and induces IL-6 neurotoxic effects (Carruthers and Suzuki, 2007). IL-6, a biomarker of chronic inflammation may explain many neurodegenerative disorders and cognitive impairment in older age (i.e., “inflammaging”, see Fard et al., 2022 for review). For example, Heyser and colleagues (1997) investigated performance in a learning task in transgenic mice that express chronically IL-6 in the brain. They found deficits in learning performance and neurodegeneration, suggesting a critical role of the proinflammatory cytokine for cognitive deficits. This was later confirmed in a human study with older adults (Weaver et al., 2002), showing that elevated baseline plasma IL-6 level enhanced the risk for subsequent decline in cognitive functions in a 2.5-year follow-up.

The open question remains, why young adults showed enhanced cognitive performance in the present study (and in basic hearing functions as reported recently by Getzmann et al., 2024). It can be assumed that the duration of latent toxoplasmosis was shorter in young adults aged between 20 and 30 years than in older participants. That is, the ratio of persons with relatively recent or current infection may be larger in the younger than in the older group which may partly explain higher IgG concentrations in the former group. *T. gondii* infection activates inflammatory responses by release of cortisol and proinflammatory cytokines IL-6 and TNF-α (Carruthers and Suzuki, 2007; Kalantari et al., 2016; Xiao et al., 2022), followed by an anti-inflammatory response to avoid autoimmune damage (Džafo et al., 2021). This mechanism might reflect a possible model of the interplay between infection, inflammation and cognitive change in a short period of time, but should be evaluated also for longer time intervals in future studies.

In summary, it could only be speculated about the underlying mechanism, why high concentrations of *T. gondii* antibodies and IL-6 and TNF-α are associated with enhanced performance of immediate and delayed recall only in younger adults, and how fast the performance diminishes with increasing age. The present results showed a temporal pattern that suggests a shift from acute to chronic *T. gondii* infection across the lifespan and reduced cognitive resources with increasing age.

It should be noted that the model proposed by Colzato et al. (2021) was developed in cross-sectional studies. However, it can only be adequately tested in a longitudinal design, ideally after controlling of time point of *T. gondii* infection, whenever possible. One longitudinal study with a five-year follow-up measure of cognitive functions in older age with toxoplasmosis was previously reported (Nimgaonkar et al., 2016). The authors showed that *T. gondii* infection (as well as other types of infections) was associated with a more rapid decline in executive function and changes in general cognitive status measured. Unfortunately, they reported a total score only, and the cognitive tests were not specified. To the best of our knowledge no further long-term studies were conducted to investigate the temporal dynamics of *T. gondii* on fluid cognition like attention, memory or executive functions in older adults.

### 4.1 Limitations, future directions and clinical implications

Overall, the effect sizes are not very strong, suggesting that the impact of *T. gondii* on cognition reveal statistical relationships, but does not necessarily indicate apparent cognitive deficits in real life. The current study is therefore exploratory in nature, and the findings are to a certain degree preliminary and need to be replicated in further studies.

There are some further limitations of the study that must be acknowledged. First, there was an age difference between the T-pos and the T-neg group. This seems to be a result of the lower prevalence of chronic *T. gondii* infection in younger adults, presumably due to higher standards of hygiene and generally less contact with contaminated water, soil or infected animals because of living in urban areas (Jones, 2001). Second, the number of cases with completed cognitive tests, *T. gondii* IgG antibody measures, and number of measured proinflammatory cytokines differed. Thus, the number of subjects with complete measurements included in the analysis of the interaction between toxoplasmosis, the cytokines and their impact on cognition was reduced. Third, the *T. gondii* antibody levels were measured separately for the two studies in two laboratories using different immunoassays. This may provide differences in assay specifications, sensitivity, and detection limits. Indeed, the mean antibody levels differed between ages 20–70 years (DVS) and 65+ (DAS), presumably due to some extremely high IgG concentrations in young and middle-aged adults. Thus, we cannot unequivocally decide whether the difference between DVS and DAS is due to the variability in individual IgG concentration or different procedures. However, the most critical analyses were performed using *T. gondii* status as seropositive and seronegative individuals irrespective the individual IgG concentration. Fourth, the present findings are based on a cross-sectional analysis. To better understand the developments of cognitive functioning as a function of *T. gondii* and/or immunological parameters, and to make causal conclusions, a longitudinal design with measures at several time points would be necessary. Indeed, the Dortmund Vital Study is conceptualized as a longitudinal study with three follow-up measures every 5 years, which will make it possible to control for cohort effects and changes in IgG antibody and proinflammatory cytokine concentrations over time. This may allow to evaluate the role of immune system in long-term chronic *T. gondii* infection in the future. In addition, further studies are required to analyze whether similar associations between chronic *T. gondii* infection and deteriorated memory functions should be confirmed in independent cohorts and age groups.

Finally, it is also important to acknowledge some clinical implications of the study. Prevention of infection with toxoplasmosis is still the best way by washing hands after working with soil, washing fruits or vegetables, sufficiently cooked meat, avoiding contact with cat feces. In case of suspected acute infection of vulnerable people, particularly pregnant women, with a *T. gondii* seronegative history, the differential diagnosis “toxoplasmosis” should be ruled out by testing. In the rare case of acute toxoplasmosis, the infection should be treated in line with the current recommendations or guidelines. Moreover, targeted screenings of *T. gondii* IgG seropositivity might be included in the standard blood test for the general population. If the IgG test reveals positive, further regular blood screenings regarding cytokine levels might be conducted to evaluate changes of the proinflammatory activity and possible risks of neuroinflammation. However, to the best of our knowledge there is a lack of effective treatment approaches to eradicate chronic infection. Existing treatments with sulfonamides in combination with pyrimethamine do not eradicate chronic infection due to the intermittent and slow rate of replication of bradyzoites (Matta et al., 2021). Therefore, larger efforts should be undertaken to develop vaccines against *T. gondii* infection (Innes et al., 2019; Zhang et al., 2022 for review) as the reported results have been replicated in several studies and fulfil the recommendation of the WHO (World Health Organisation, 1992).

## 5 Conclusions

The present study evaluated changes of cognitive functions across the adult life span and the role of latent *T. gondii* infection and inflammation for the cognitive trajectories. Generally, the data supported a model proposing direct and indirect effects of *T. gondii* on cognitive functions, especially memory functions and to lower extent executive functions due to acute infection and inflammation, and long-term effects of chronic inflammation leading to neurodegeneration and accelerated cognitive decline in older adults.

## CRediT authorship contribution statement

**Patrick D. Gajewski:** Writing – review & editing, Writing – original draft, Visualization, Methodology, Formal analysis, Data curation, Conceptualization. **Peter Bröde:** Writing – review & editing, Methodology. **Maren Claus:** Writing – review & editing, Methodology, Investigation, Data curation. **Klaus Golka:** Writing – review & editing, Conceptualization. **Jan G. Hengstler:** Writing – review & editing, Project administration, Conceptualization. **Jörg Reinders:** Data curation. **Carsten Watzl:** Writing – review & editing, Data curation. **Edmund Wascher:** Supervision, Resources, Project administration. **Stephan Getzmann:** Writing – review & editing, Supervision, Project administration, Methodology, Conceptualization.

## Funding

The Dortmund Vital Study is funded by the institute's budget. Thus, the study design, collection, management, analysis, interpretation of data, writing of the report, and the decision to submit the report for publication are not influenced or biased by any sponsor. Aditionally, the study has been partly supported by the Leibniz Research Alliance “Resilient Ageing” (Project number: LFV-2021-2-LIR).

## Declaration of competing interest

The authors declare that they have no interests to declare.

## Data Availability

Data will be made available on request.
