# Supplementary material for: Changes of cognitive functions and proinflammatory cytokines across the lifespan in latent Toxoplasma gondii infection
Source: Brain Behav Immun Health. 2025 Sep 15;49:101105. doi: 10.1016/j.bbih.2025.101105 (PMC12464602; doi:10.1016/j.bbih.2025.101105)
Supplement: Multimedia component 1 [file mmc1.docx]

Supplementary Material

**Changes of cognitive functions and proinflammatory cytokines across the lifespan in latent *Toxoplasma gondii* infection**

**Table S1**. Descriptive statistics of the neuropsychological test scores for the whole sample.

| **Variable** | ***n*** | ***M*** | ***SD*** | ***min*** | ***max*** |
| --- | --- | --- | --- | --- | --- |
| Digit span forward (n correct) | 691 | 7.50 | 2.02 | 3 | 13 |
| Digit span backward (n correct) | 691 | 6.51 | 2.00 | 2 | 13 |
| Digit span total (n correct) | 691 | 13.99 | 3.56 | 4 | 25 |
| VLMT Ʃ 1-5: Learning span (n correct) | 638 | 52.35 | 10.92 | 22 | 74 |
| VLMT 6: Post-interference (n correct) | 635 | 11.00 | 3.09 | 2 | 15 |
| VLMT 7: Delayed recall (n correct) | 637 | 11.08 | 3.15 | 2 | 15 |
| VLMT W-F: Delayed recognition (n correct) | 637 | 13.02 | 2.71 | -5 | 15 |
| DST: Psychomotor speed (n correct) | 639 | 57.18 | 13.34 | 0 | 93 |
| Stroop 1 (time in sec) | 627 | 13.80 | 2.46 | 9 | 26 |
| Stroop 2 (time in sec) | 627 | 20.36 | 3.68 | 12 | 45 |
| Stroop 3 (time in sec) | 627 | 33.33 | 9.31 | 17 | 105 |
| Stroop 3-2: interference (time in sec) | 627 | 12.97 | 7.76 | -3 | 80 |
| TMT A (time in sec) | 629 | 27.60 | 10.88 | 10 | 82 |
| TMT B (time in sec) | 629 | 63.86 | 29.44 | 15 | 264 |
| TMT B-A: Cognitive flexibility (time in sec) | 629 | 36.26 | 24.08 | -11 | 214 |
| LPS 3: Logical reasoning (n correct) | 542 | 27.42 | 5.68 | 6 | 40 |
| LPS 6: Verbal fluency (n correct) | 596 | 37.62 | 7.91 | 13 | 61 |
| LPS7: Mental rotation (n correct) | 540 | 21.80 | 7.69 | 2 | 40 |
| CFQ: Cognitive failures (n correct) | 683 | 29.90 | 11.48 | 4 | 82 |

**Table S2**. Descriptive statistics of the concentrations of the proinflammatory cytokines in serum in the whole subsample with the available cytokine measures.

| **Variable** | ***n*** | ***M*** | ***SD*** | ***min*** | ***max*** |
| --- | --- | --- | --- | --- | --- |
| IL-6 (pg/ml) | 296 | 4.45 | 7.49 | 0.06 | 75.15 |
| IL-8 (pg/ml) | 293 | 10.77 | 12.50 | 0.28 | 82.96 |
| IL-18 (pg/ml) | 286 | 58.62 | 49.57 | 1.66 | 278.64 |
| TNF- α (pg/ml) | 273 | 1.88 | 3.27 | 0.04 | 35.10 |

Table S3. Results of the moderator analyses with the total score of trial 7 of the VLMT (VLMT-7) as dependent variable, age as independent variable, and *T. gondii* and log10 transformed IL-6, IL-8, IL-18 and TNF-α as moderator variables. Δ*R²* indicates changes of the *R^2^* by the three-way interaction term. Bold text indicates significance.

| Outcome: **VLMT-7** | *b ^a)^* | *SE ^a)^* | *t ^a)^* | *p ^a)^* |  |
| --- | --- | --- | --- | --- | --- |
| Moderator: **IL-6** | | | | | |
| Intercept | 16.547 | 8.816 | 1.877 | .064 |  |
| *T. gondii* | .256 | 3.840 | .067 | .947 |  |
| Age | -.206 | .166 | -1.242 | .217 |  |
| IL-6 | .041 | .074 | .552 | .582 |  |
| *T. gondii* x Age | -21.640 | 21.082 | -1.026 | .307 |  |
| *T. gondii* x IL-6 | 11.083 | 8.887 | 1.247 | .215 |  |
| Age x IL-6 | .465 | .326 | 1.426 | .157 |  |
| *T. gondii* x Age x IL-6 *^b)^* | -.239 | .141 | -1.700 | .092 |  |
| *N* = 106; *R* = .687; *R^2^* = .472; *F*(7, 98) = 12.53; *p* < .001; Δ*R²* = .016 *^b)^* | | | | | |
| Moderator: **IL-8** | | | | | |
| Intercept | 10.547 | 15.832 | .666 | .507 |  |
| *T. gondii* | 1.048 | 6.507 | .161 | .872 |  |
| Age | -.152 | .295 | -.514 | .608 |  |
| IL-8 | .057 | .126 | .451 | .653 |  |
| *T. gondii* x Age | -2.635 | 20.448 | -.129 | .898 |  |
| *T. gondii* x IL-8 | 3.827 | 8.717 | .439 | .662 |  |
| Age x IL-8 | .173 | .332 | .522 | .603 |  |
| *T. gondii* x Age x IL-8 *^b)^* | -.134 | .144 | -.932 | .354 |  |
| *N* = 104; *R* = .694; *R^2^* = .482; *F*(7, 96) = 12.77; *p* < .001, Δ*R²* = .005 *^b)^* | | | | |  |
| Moderator: **IL-18** | | | | |  |
| Intercept | 9.170 | 49.825 | .184 | .854 |  |
| *T. gondii* | 7.308 | 21.615 | .338 | .736 |  |
| Age | .005 | .726 | .006 | .995 |  |
| IL-18 | -.121 | .317 | -.382 | .704 |  |
| *T. gondii* x Age | .361 | 27.369 | .013 | .990 |  |
| *T. gondii* x IL-18 | -1.984 | 11.867 | -.167 | .868 |  |
| Age x IL-18 | -.014 | .407 | -.035 | .972 |  |
| *T. gondii* x Age x IL-18 *^b)^*  *^b)^* | .038 | .178 | .213 | .831 |  |
| *N* = 99; *R* = .703; *R^2^* = .495; *F*(7, 91) = 12.73; *p* < .001, Δ*R²* = .000 *^b)^* | | | | |  |
| Moderator: **TNF-α** | | | | |  |
| Intercept | 2.772 | 8.491 | .326 | .745 |  |
| *T. gondii* | 6.853 | 3.780 | 1.813 | .073 |  |
| Age | .085 | .136 | .622 | .535 |  |
| TNF-α | -.100 | .061 | -1.638 | .105 |  |
| *T. gondii* x Age | -45.525 | 19.418 | **-2.344** | **.021** |  |
| *T. gondii* x TNF-α | 19.308 | 8.162 | **2.365** | **.020** |  |
| Age x TNF-α | .700 | .313 | **2.235** | **.028** |  |
| *T. gondii* x Age x TNF-α *^b)^* | -.307 | .134 | **-2.294** | **.024** |  |
| *N* = 102; *R* = .685; *R^2^* = .470; *F*(7, 94) = 11.93; *p* < .001, Δ*R²* = .030 *^b)^* | | | | |  |
| *^a)^ regression coefficients (b) with standard errors (SE), t- and p-values* | | | | |  |
| *^b)^ ΔR²: change in R^2^ due to the three-way interaction term* | | | | |  |

Table S4. Results of the moderator analyses with the total score of trial W-F of the VLMT (VLMT-W-F) as dependent variable, age as independent variable, and *T. gondii* and log1010 transformed IL-6, IL-8, IL-18 and TNF-α as moderator variables. Δ*R²* indicates changes of the *R^2^* by the three-way interaction term.Bold text indicates significance.

| Outcome: **VLMT-W-F** | *b ^a)^* | *SE ^a)^* | *t ^a)^* | *p ^a)^* |  |
| --- | --- | --- | --- | --- | --- |
| Moderator: **IL-6** | | | | | |
| Intercept | 15.387 | 7.193 | 2.139 | .035 |  |
| *T. gondii* | -.044 | 3.133 | -.014 | .989 |  |
| Age | -.024 | .135 | -.179 | .859 |  |
| IL-6 | -.005 | .061 | -.090 | .928 |  |
| *T. gondii* x Age | -1.040 | 17.200 | -.060 | .952 |  |
| *T. gondii* x IL-6 | .648 | 7.250 | .089 | .929 |  |
| Age x IL-6 | .053 | .266 | .198 | .843 |  |
| *T. gondii* x Age x IL-6 *^b)^* | -.030 | .115 | -.263 | .793 |  |
| *N* = 106; *R* = .324; *R^2^* = .105; *F*(7, 98) = 1.64; *p* = .132; Δ*R²* = .001 *^b)^* | | | | | |
| Moderator: **IL-8** | | | | | |
| Intercept | 17.796 | 12.509 | 1.423 | .158 |  |
| *T. gondii* | -2.058 | 5.141 | -.400 | .690 |  |
| Age | -.228 | .233 | -.977 | .331 |  |
| IL-8 | .107 | .099 | 1.078 | .284 |  |
| *T. gondii* x Age | 4.905 | 16.157 | .304 | .762 |  |
| *T. gondii* x IL-8 | -1.369 | 6.888 | -.199 | .843 |  |
| Age x IL-8 | .112 | .263 | .427 | .670 |  |
| *T. gondii* x Age x IL-8 *^b)^* | -.073 | .114 | -.642 | .523 |  |
| *N* = 104; *R* = .430; *R^2^* = .185; *F*(7, 96) = 3.11; *p* < .005, Δ*R²* = .003 *^b)^* | | | | |  |
| Moderator: **IL-18** | | | | |  |
| Intercept | 24.588 | 40.946 | .600 | .550 |  |
| *T. gondii* | -1.582 | 17.763 | -.089 | .929 |  |
| Age | -.230 | .597 | -.385 | .701 |  |
| IL-18 | .042 | .260 | .162 | .872 |  |
| *T. gondii* x Age | -8.259 | 22.492 | -.367 | .714 |  |
| *T. gondii* x IL-18 | 2.274 | 9.752 | .233 | .816 |  |
| Age x IL-18 | .202 | .334 | .604 | .548 |  |
| *T. gondii* x Age x IL-18 *^b)^*  *^b)^* | -.066 | .146 | -.451 | .653 |  |
| *N* = 99; *R* = .403; *R^2^* = .163; *F*(7, 91) = 2.52; *p* < .05, Δ*R²* = .002 *^b)^* | | | | |  |
| Moderator: **TNF-α** | | | | |  |
| Intercept | 13.473 | 6.956 | 1.937 | .056 |  |
| *T. gondii* | .890 | 3.097 | .287 | .774 |  |
| Age | .026 | .112 | .229 | .819 |  |
| TNF-α | -.031 | .050 | -.625 | .534 |  |
| *T. gondii* x Age | -8.506 | 15.908 | -.535 | .594 |  |
| *T. gondii* x TNF-α | 3.081 | 6.687 | .461 | .646 |  |
| Age x TNF-α | .134 | .257 | .521 | .604 |  |
| *T. gondii* x Age x TNF-α *^b)^* | -.050 | .110 | -.458 | .648 |  |
| *N* = 102; *R* = .320; *R^2^* = .102; *F*(7, 94) = 4.58; *p* = .167, Δ*R²* = .002 *^b)^* | | | | |  |
| *^a)^ regression coefficients (b) with standard errors (SE), t- and p-values* | | | | |  |
| *^b)^ ΔR²: change in R^2^ due to the three-way interaction term* | | | | |  |

Table S5. Results of the moderator analyses with the digit span forward (DS-F) as dependent variable, age as independent variable, and *T. gondii* and log10 transformed IL-6, IL-8, IL-18 and TNF-α as moderator variables. Δ*R²* indicates changes of the *R^2^* by the three-way interaction term.Bold text indicates significance.

| Outcome: **DS-F** | *b ^a)^* | *SE ^a)^* | *t ^a)^* | *p ^a)^* |  |
| --- | --- | --- | --- | --- | --- |
| Moderator: **IL-6** | | | | | |
| Intercept | 14.116 | 6.751 | 2.091 | .039 |  |
| *T. gondii* | -2.263 | 2.926 | -.774 | .441 |  |
| Age | -.107 | .125 | -.857 | .393 |  |
| IL-6 | .033 | .056 | .594 | .554 |  |
| *T. gondii* x Age | -31.524 | 15.268 | **-2.065** | **.041** |  |
| *T. gondii* x IL-6 | 13.836 | 6.476 | **2.137** | **.035** |  |
| Age x IL-6 | .435 | .238 | 1.828 | .070 |  |
| *T. gondii* x Age x IL-6 *^b)^* | -.187 | .103 | -1.813 | .073 |  |
| *N* = 110; *R* = .310; *R^2^* = .096; *F*(7, 102) = 1.55; *p* = .158; Δ*R²* = .029 *^b)^* | | | | | |
| Moderator: **IL-8** | | | | | |
| Intercept | 16.298 | 11.822 | 1.379 | .171 |  |
| *T. gondii* | -4.137 | 4.866 | -.850 | .397 |  |
| Age | -.159 | .221 | -.722 | .472 |  |
| IL-8 | .072 | .094 | .763 | .447 |  |
| *T. gondii* x Age | -12.026 | 15.415 | -.780 | .437 |  |
| *T. gondii* x IL-8 | 5.970 | 6.577 | .908 | .366 |  |
| Age x IL-8 | .185 | .250 | .739 | .462 |  |
| *T. gondii* x Age x IL-8 *^b)^* | -.091 | .109 | -.838 | .404 |  |
| *N* = 108; *R* = .182; *R^2^* = .033; *F*(7, 100) = 0.48; *p* = .840, Δ*R²* = .007 *^b)^* | | | | |  |
| Moderator: **IL-18** | | | | |  |
| Intercept | 9.170 | 49.825 | .184 | .854 |  |
| *T. gondii* | 7.308 | 21.615 | .338 | .736 |  |
| Age | .005 | .726 | .006 | .995 |  |
| IL-18 | -.121 | .317 | -.382 | .704 |  |
| *T. gondii* x Age | .361 | 27.369 | .013 | .990 |  |
| *T. gondii* x IL-18 | -1.984 | 11.867 | -.167 | .868 |  |
| Age x IL-18 | -.014 | .407 | -.035 | .972 |  |
| *T. gondii* x Age x IL-18 *^b)^*  *^b)^* | .038 | .178 | .213 | .831 |  |
| *N* = 104; *R* = .303; *R^2^* = .092; *F*(7, 96) = 1.38; *p* = .220, Δ*R²* = .000 *^b)^* | | | | |  |
| Moderator: **TNF-α** | | | | |  |
| Intercept | -4.307 | 40.980 | -.105 | .917 |  |
| *T. gondii* | .475 | 17.591 | .027 | .978 |  |
| Age | .174 | .596 | .292 | .771 |  |
| TNF-α | -.012 | .257 | -.046 | .963 |  |
| *T. gondii* x Age | 9.347 | 22.136 | .422 | .674 |  |
| *T. gondii* x TNF-α | -1.268 | 9.525 | -.133 | .894 |  |
| Age x TNF-α | -.156 | .327 | -.477 | .634 |  |
| *T. gondii* x Age x TNF-α *^b)^* | .028 | .142 | .196 | .845 |  |
| *N* = 107; *R* = .311; *R^2^* = .097; *F*(7, 99) = 1.51; *p* = .172, Δ*R²* = .006 *^b)^* | | | | |  |
| *^a)^ regression coefficients (b) with standard errors (SE), t- and p-values* | | | | |  |
| *^b)^ ΔR²: change in R^2^ due to the three-way interaction term* | | | | |  |

Table S6. Results of the moderator analyses with the digit span backward (DS-B) as dependent variable, age as independent variable, and *T. gondii* and log10 transformed IL-6, IL-8, IL-18 and TNF-α as moderator variables. Δ*R²* indicates changes of the *R^2^* by the three-way interaction term.Bold text indicates significance.

| Outcome: **DS-B** | *b ^a)^* | *SE ^a)^* | *t ^a)^* | *p ^a)^* |  |
| --- | --- | --- | --- | --- | --- |
| Moderator: **IL-6** | | | | | |
| Intercept | 12.320 | 5.444 | 2.263 | .026 |  |
| *T. gondii* | -1.254 | 2.381 | -.526 | .600 |  |
| Age | -.087 | .102 | -.849 | .398 |  |
| IL-6 | .012 | .046 | .263 | .793 |  |
| *T. gondii* x Age | -4.250 | 12.845 | -.331 | .741 |  |
| *T. gondii* x IL-6 | 1.384 | 5.450 | .254 | .800 |  |
| Age x IL-6 | .009 | .200 | .045 | .964 |  |
| *T. gondii* x Age x IL-6 *^b)^* | .007 | .087 | .081 | .936 |  |
| *N* = 110; *R* = .459; *R^2^* = .211; *F*(7, 102) = 3.88; *p* < .001; Δ*R²* = .000 *^b)^* | | | | | |
| Moderator: **IL-8** | | | | | |
| Intercept | 20.988 | 9.587 | 2.189 | .031 |  |
| *T. gondii* | -4.584 | 3.947 | -1.161 | .248 |  |
| Age | -.228 | .179 | -1.275 | .205 |  |
| IL-8 | .069 | .076 | .905 | .368 |  |
| *T. gondii* x Age | -7.600 | 12.428 | -.612 | .542 |  |
| *T. gondii* x IL-8 | 2.150 | 5.296 | .406 | .686 |  |
| Age x IL-8 | .097 | .202 | .479 | .633 |  |
| *T. gondii* x Age x IL-8 *^b)^* | -.025 | .088 | -.283 | .778 |  |
| *N* = 104; *R* = .694; *R^2^* = .482; *F*(7, 96) = 12.77; *p* < .001, Δ*R²* = .005 *^b)^* | | | | |  |
| Moderator: **IL-18** | | | | |  |
| Intercept | -25.679 | 32.270 | -.796 | .428 |  |
| *T. gondii* | 15.198 | 14.000 | 1.086 | .280 |  |
| Age | .380 | .470 | .807 | .422 |  |
| IL-18 | -.185 | .205 | -.903 | .369 |  |
| *T. gondii* x Age | 21.353 | 17.707 | 1.206 | .231 |  |
| *T. gondii* x IL-18 | -9.356 | 7.681 | -1.218 | .226 |  |
| Age x IL-18 | -.273 | .263 | -1.036 | .303 |  |
| *T. gondii* x Age x IL-18 *^b)^*  *^b)^* | .118 | .115 | 1.030 | .306 |  |
| *N* = 104; *R* = .472; *R^2^* = .222 *F*(7, 96) = 3.92; *p* < .001, Δ*R²* = .009 *^b)^* | | | | |  |
| Moderator: **TNF-α** | | | | |  |
| Intercept | 15.763 | 5.225 | 3.017 | .003 |  |
| *T. gondii* | -2.968 | 2.332 | -1.273 | .206 |  |
| Age | -.177 | .084 | **-2.109** | **.037** |  |
| TNF-α | .057 | .038 | 1.494 | .138 |  |
| *T. gondii* x Age | 7.623 | 11.745 | .649 | .518 |  |
| *T. gondii* x TNF-α | -2.747 | 4.969 | -.553 | .582 |  |
| Age x TNF-α | -.064 | .191 | -.333 | .740 |  |
| *T. gondii* x Age x TNF-α *^b)^* | .021 | .082 | .256 | .799 |  |
| *N* = 107; *R* = .473; *R^2^* = .224; *F*(7, 99) = 4.07; *p* < .001, Δ*R²* = .001 *^b)^* | | | | |  |
| *^a)^ regression coefficients (b) with standard errors (SE), t- and p-values* | | | | |  |
| *^b)^ ΔR²: change in R^2^ due to the three-way interaction term* | | | | |  |

Table S7. Results of the moderator analyses with the Stroop 2 as dependent variable, age as independent variable, and *T. gondii* and log10 transformed IL-6, IL-8, IL-18 and TNF-α as moderator variables. Δ*R²* indicates changes of the *R^2^* by the three-way interaction term.Bold text indicates significance.

| Outcome: **Stroop 2** | *b ^a)^* | *SE ^a)^* | *t ^a)^* | *p ^a)^* |  |
| --- | --- | --- | --- | --- | --- |
| Moderator: **IL-6** | | | | | |
| Intercept | 22.204 | 14.843 | 1.496 | .138 |  |
| *T. gondii* | -3.044 | 6.714 | -.453 | .651 |  |
| Age | -.022 | .268 | -.083 | .934 |  |
| IL-6 | .063 | .124 | .508 | .613 |  |
| *T. gondii* x Age | 12.557 | 38.911 | .323 | .748 |  |
| *T. gondii* x IL-6 | -4.402 | 17.148 | -.257 | .798 |  |
| Age x IL-6 | -.082 | .603 | -.135 | .893 |  |
| *T. gondii* x Age x IL-6 *^b)^* | -.001 | .270 | -.002 | .998 |  |
| *N* = 104; *R* = .386; *R^2^* = .149; *F*(7, 96) = 2.40; *p* < .05; Δ*R²* = .000 *^b)^* | | | | | |
| Moderator: **IL-8** | | | | | |
| Intercept | 4.233 | 22.245 | .190 | .849 |  |
| *T. gondii* | 3.069 | 9.292 | .330 | .742 |  |
| Age | .117 | .414 | .282 | .778 |  |
| IL-8 | .032 | .179 | .182 | .856 |  |
| *T. gondii* x Age | 32.388 | 28.085 | 1.153 | .252 |  |
| *T. gondii* x IL-8 | -11.604 | 12.022 | -.965 | .337 |  |
| Age x IL-8 | -.303 | .459 | -.661 | .510 |  |
| *T. gondii* x Age x IL-8 *^b)^* | .078 | .200 | .392 | .696 |  |
| *N* = 102; *R* = .359; *R^2^* = .129; *F*(7, 94) = 1.98; *p* = .065, Δ*R²* = .001 *^b)^* | | | | |  |
| Moderator: **IL-18** | | | | |  |
| Intercept | 126.345 | 85.611 | 1.476 | .144 |  |
| *T. gondii* | -48.197 | 38.508 | -1.252 | .214 |  |
| Age | -1.196 | 1.235 | -.968 | .336 |  |
| IL-18 | .545 | .556 | .980 | .330 |  |
| *T. gondii* x Age | -52.130 | 45.477 | -1.146 | .255 |  |
| *T. gondii* x IL-18 | 22.794 | 20.341 | 1.121 | .265 |  |
| Age x IL-18 | .555 | .664 | .835 | .406 |  |
| *T. gondii* x Age x IL-18 *^b)^*  *^b)^* | -.234 | .298 | -.784 | .435 |  |
| *N* = 97; *R* = .393; *R^2^* = .154; *F*(7, 89) = 2.32; *p* < .05, Δ*R²* = .006 *^b)^* | | | | |  |
| Moderator: **TNF-α** | | | | |  |
| Intercept | 28.995 | 11.074 | 2.618 | .010 |  |
| *T. gondii* | -5.221 | 4.937 | -1.057 | .293 |  |
| Age | -.144 | .178 | -.809 | .421 |  |
| TNF-α | .092 | .080 | 1.146 | .255 |  |
| *T. gondii* x Age | -21.492 | 36.834 | -.583 | .561 |  |
| *T. gondii* x TNF-α | 9.337 | 16.417 | .569 | .571 |  |
| Age x TNF-α | .692 | .582 | 1.189 | .237 |  |
| *T. gondii* x Age x TNF-α *^b)^* | -.312 | .262 | -1.188 | .238 |  |
| *N* = 100; *R* = .419; *R^2^* = .176; *F*(7, 92) = 2.80; *p* < .05, Δ*R²* = .013 *^b)^* | | | | |  |
| *^a)^ regression coefficients (b) with standard errors (SE), t- and p-values* | | | | |  |
| *^b)^ ΔR²: change in R^2^ due to the three-way interaction term* | | | | |  |

Table S8. Results of the moderator analyses with the Stroop 3 as dependent variable, age as independent variable, and *T. gondii* and log10 transformed IL-6, IL-8, IL-18 and TNF-α as moderator variables. Δ*R²* indicates changes of the *R^2^* by the three-way interaction term.Bold text indicates significance.

| Outcome: **Stroop 3** | *b ^a)^* | *SE ^a)^* | *t ^a)^* | *p ^a)^* |  |
| --- | --- | --- | --- | --- | --- |
| Moderator: **IL-6** | | | | | |
| Intercept | 19.405 | 33.277 | .583 | .561 |  |
| *T. gondii* | -3.298 | 15.052 | -.219 | .827 |  |
| Age | .396 | .601 | .659 | .511 |  |
| IL-6 | .016 | .277 | .057 | .954 |  |
| *T. gondii* x Age | 65.715 | 87.237 | .753 | .453 |  |
| *T. gondii* x IL-6 | -24.881 | 38.446 | -.647 | .519 |  |
| Age x IL-6 | -.817 | 1.351 | -.605 | .547 |  |
| *T. gondii* x Age x IL-6 *^b)^* | .301 | .605 | .497 | .620 |  |
| *N* = 104; *R* = .655; *R^2^* = .429; *F*(7, 96) = 10.30; *p* < .001; Δ*R²* = .001 *^b)^* | | | | | |
| Moderator: **IL-8** | | | | | |
| Intercept | 26.448 | 48.742 | .543 | .589 |  |
| *T. gondii* | -5.361 | 20.360 | -.263 | .793 |  |
| Age | -.128 | .908 | -.141 | .888 |  |
| IL-8 | .203 | .392 | .517 | .606 |  |
| *T. gondii* x Age | 22.360 | 61.540 | .363 | .717 |  |
| *T. gondii* x IL-8 | -8.610 | 26.342 | -.327 | .745 |  |
| Age x IL-8 | .063 | 1.005 | .063 | .950 |  |
| *T. gondii* x Age x IL-8 *^b)^* | -.019 | .438 | -.043 | .966 |  |
| *N* = 102; *R* = .655; *R^2^* = .430; *F*(7, 94) = 10.11; *p* < .001, Δ*R²* = .000 *^b)^* | | | | |  |
| Moderator: **IL-18** | | | | |  |
| Intercept | 202.544 | 184.401 | 1.098 | .275 |  |
| *T. gondii* | -83.141 | 82.945 | -1.002 | .319 |  |
| Age | -1.573 | 2.660 | -.591 | .556 |  |
| IL-18 | .861 | 1.198 | .719 | .474 |  |
| *T. gondii* x Age | -84.453 | 97.956 | -.862 | .391 |  |
| *T. gondii* x IL-18 | 37.227 | 43.814 | .850 | .398 |  |
| Age x IL-18 | .810 | 1.431 | .566 | .573 |  |
| *T. gondii* x Age x IL-18 *^b)^*  *^b)^* | -.352 | .641 | -.549 | .584 |  |
| *N* = 97; *R* = .687; *R^2^* = .472; *F*(7, 89) = 11.38; *p* < .001, Δ*R²* = .002 *^b)^* | | | | |  |
| Moderator: **TNF-α** | | | | |  |
| Intercept | 34.537 | 24.934 | 1.385 | .169 |  |
| *T. gondii* | -8.857 | 11.115 | -.797 | .428 |  |
| Age | .107 | .401 | .267 | .790 |  |
| TNF-α | .128 | .181 | .705 | .483 |  |
| *T. gondii* x Age | -84.031 | 82.931 | -1.013 | .314 |  |
| *T. gondii* x TNF-α | 41.930 | 36.962 | 1.134 | .260 |  |
| Age x TNF-α | 1.762 | 1.310 | 1.345 | .182 |  |
| *T. gondii* x Age x TNF-α *^b)^* | -.846 | .591 | -1.432 | .156 |  |
| *N* = 100; *R* = .665; *R^2^* = .442; *F*(7, 92) = 10.41; *p* < .001, Δ*R²* = .012 *^b)^* | | | | |  |
| *^a)^ regression coefficients (b) with standard errors (SE), t- and p-values* | | | | |  |
| *^b)^ ΔR²: change in R^2^ due to the three-way interaction term* | | | | |  |

Table S9. Results of the moderator analyses with the TMT-A as dependent variable, age as independent variable, and *T. gondii* and log10 transformed IL-6, IL-8, IL-18 and TNF-α as moderator variables. Δ*R²* indicates changes of the *R^2^* by the three-way interaction term.Bold text indicates significance.

| Outcome: **TMT-A** | *b ^a)^* | *SE ^a)^* | *t ^a)^* | *p ^a)^* |  |
| --- | --- | --- | --- | --- | --- |
| Moderator: **IL-6** | | | | | |
| Intercept | 46.761 | 32.699 | 1.430 | .156 |  |
| *T. gondii* | -14.180 | 14.260 | -.994 | .323 |  |
| Age | -.105 | .622 | -.170 | .866 |  |
| IL-6 | .176 | .279 | .631 | .529 |  |
| *T. gondii* x Age | -91.113 | 80.492 | -1.132 | .261 |  |
| *T. gondii* x IL-6 | 37.883 | 34.039 | 1.113 | .269 |  |
| Age x IL-6 | 1.278 | 1.275 | 1.002 | .319 |  |
| *T. gondii* x Age x IL-6 *^b)^* | -.524 | .551 | -.951 | .344 |  |
| *N* = 102; *R* = .514; *R^2^* = .264; *F*(7, 94) = 4.82; *p* < .001; Δ*R²* = .007 *^b)^* | | | | | |
| Moderator: **IL-8** | | | | | |
| Intercept | 58.323 | 59.416 | .982 | .329 |  |
| *T. gondii* | -20.147 | 24.502 | -.822 | .413 |  |
| Age | -.976 | 1.139 | -.857 | .394 |  |
| IL-8 | .542 | .488 | 1.111 | .269 |  |
| *T. gondii* x Age | -.604 | 76.989 | -.008 | .994 |  |
| *T. gondii* x IL-8 | -.199 | 32.733 | -.006 | .995 |  |
| Age x IL-8 | .543 | 1.272 | .427 | .671 |  |
| *T. gondii* x Age x IL-8 *^b)^* | -.200 | .550 | -.363 | .717 |  |
| *N* = 100; *R* = .553; *R^2^* = .306; *F*(7, 92) = 5.78; *p* < .001, Δ*R²* = .001 *^b)^* | | | | |  |
| Moderator: **IL-18** | | | | |  |
| Intercept | 146.005 | 187.387 | .779 | .438 |  |
| *T. gondii* | -68.431 | 81.402 | -.841 | .403 |  |
| Age | -1.069 | 2.730 | -.391 | .696 |  |
| IL-18 | .745 | 1.192 | .625 | .534 |  |
| *T. gondii* x Age | -51.618 | 102.490 | -.504 | .616 |  |
| *T. gondii* x IL-18 | 27.665 | 44.460 | .622 | .535 |  |
| Age x IL-18 | .378 | 1.522 | .248 | .805 |  |
| *T. gondii* x Age x IL-18 *^b)^*  *^b)^* | -.230 | .665 | -.346 | .730 |  |
| *N* = 95; *R* = .536; *R^2^* = .287; *F*(7, 87) = 5.06; *p* < .001, Δ*R²* = .001 *^b)^* | | | | |  |
| Moderator: **TNF-α** | | | | |  |
| Intercept | 29.312 | 31.972 | .917 | .362 |  |
| *T. gondii* | -7.594 | 14.306 | -.531 | .597 |  |
| Age | .144 | .523 | .276 | .783 |  |
| TNF-α | .091 | .236 | .385 | .701 |  |
| *T. gondii* x Age | -5.234 | 71.748 | -.073 | .942 |  |
| *T. gondii* x TNF-α | 7.829 | 30.263 | .259 | .796 |  |
| Age x TNF-α | .101 | 1.167 | .087 | .931 |  |
| *T. gondii* x Age x TNF-α *^b)^* | -.147 | .501 | -.293 | .770 |  |
| *N* = 98; *R* = .514; *R^2^* = .264; *F*(7, 90) = 4.60; *p* < .001, Δ*R²* = .001 *^b)^* | | | | |  |
| *^a)^ regression coefficients (b) with standard errors (SE), t- and p-values* | | | | |  |
| *^b)^ ΔR²: change in R^2^ due to the three-way interaction term* | | | | |  |

Table S10. Results of the moderator analyses with the TMT-B as dependent variable, age as independent variable, and *T. gondii* and log10 transformed IL-6, IL-8, IL-18 and TNF-α as moderator variables. Δ*R²* indicates changes of the *R^2^* by the three-way interaction term.Bold text indicates significance.

| Outcome: **TMT-B** | *b ^a)^* | *SE ^a)^* | *t ^a)^* | *p ^a)^* |  |
| --- | --- | --- | --- | --- | --- |
| Moderator: **IL-6** | | | | | |
| Intercept | 99.739 | 84.605 | 1.179 | .241 |  |
| *T. gondii* | -39.409 | 36.895 | -1.068 | .288 |  |
| Age | -.655 | 1.609 | -.407 | .685 |  |
| IL-6 | .773 | .721 | 1.072 | .286 |  |
| *T. gondii* x Age | -13.538 | 208.262 | -.065 | .948 |  |
| *T. gondii* x IL-6 | 2.904 | 88.072 | .033 | .974 |  |
| Age x IL-6 | .631 | 3.299 | .191 | .849 |  |
| *T. gondii* x Age x IL-6 *^b)^* | -.180 | 1.425 | -.126 | .900 |  |
| *N* = 102; *R* = .615; *R^2^* = .379; *F*(7, 94) = 8.18; *p* < .001; Δ*R²* = .000 *^b)^* | | | | | |
| Moderator: **IL-8** | | | | | |
| Intercept | -58.743 | 156.219 | -.376 | .708 |  |
| *T. gondii* | 26.325 | 64.422 | .409 | .684 |  |
| Age | 2.654 | 2.994 | .887 | .378 |  |
| IL-8 | -.667 | 1.282 | -.521 | .604 |  |
| *T. gondii* x Age | 166.987 | 202.421 | .825 | .412 |  |
| *T. gondii* x IL-8 | -70.711 | 86.062 | -.822 | .413 |  |
| Age x IL-8 | -3.150 | 3.344 | -.942 | .349 |  |
| *T. gondii* x Age x IL-8 *^b)^* | 1.429 | 1.445 | .989 | .325 |  |
| *N* = 100; *R* = .622; *R^2^* = .387; *F*(7, 92) = 8.31; *p* < .001, Δ*R²* = .007 *^b)^* | | | | |  |
| Moderator: **IL-18** | | | | |  |
| Intercept | 556.842 | 477.461 | 1.166 | .247 |  |
| *T. gondii* | -269.969 | 207.412 | -1.302 | .196 |  |
| Age | -6.909 | 6.957 | -.993 | .323 |  |
| IL-18 | 4.042 | 3.038 | 1.331 | .187 |  |
| *T. gondii* x Age | -264.700 | 261.142 | -1.014 | .314 |  |
| *T. gondii* x IL-18 | 132.762 | 113.285 | 1.172 | .244 |  |
| Age x IL-18 | 3.881 | 3.879 | 1.001 | .320 |  |
| *T. gondii* x Age x IL-18 *^b)^*  *^b)^* | -1.991 | 1.695 | -1.175 | .243 |  |
| *N* = 95; *R* = .634; *R^2^* = .402; *F*(7, 87) = 8.36; *p* < .001, Δ*R²* = .009 *^b)^* | | | | |  |
| Moderator: **TNF-α** | | | | |  |
| Intercept | 79.790 | 83.697 | .953 | .343 |  |
| *T. gondii* | -32.863 | 37.451 | -.877 | .383 |  |
| Age | -.158 | 1.369 | -.115 | .909 |  |
| TNF-α | .623 | .619 | 1.007 | .317 |  |
| *T. gondii* x Age | -22.689 | 187.824 | -.121 | .904 |  |
| *T. gondii* x TNF-α | 19.428 | 79.223 | .245 | .807 |  |
| Age x TNF-α | .972 | 3.055 | .318 | .751 |  |
| *T. gondii* x Age x TNF-α *^b)^* | -.590 | 1.312 | -.450 | .654 |  |
| *N* = 98; *R* = .609; *R^2^* = .371; *F*(7, 90) = 7.58; *p* < .001, Δ*R²* = .001 *^b)^* | | | | |  |
| *^a)^ regression coefficients (b) with standard errors (SE), t- and p-values* | | | | |  |
| *^b)^ ΔR²: change in R^2^ due to the three-way interaction term* | | | | |  |

Table S11. Results of the moderator analyses with the Digit Symbol Test (DST) as dependent variable, age as independent variable, and *T. gondii* and log10 transformed IL-6, IL-8, IL-18 and TNF-α as moderator variables. Δ*R²* indicates changes of the *R^2^* by the three-way interaction term. Bold text indicates significance.

| Outcome: **DST** | *b ^a)^* | *SE ^a)^* | *t ^a)^* | *p ^a)^* |  |
| --- | --- | --- | --- | --- | --- |
| Moderator: **IL-6** | | | | | |
| Intercept | 89.196 | 32.264 | 2.765 | .007 |  |
| *T. gondii* | -3.547 | 14.050 | -.252 | .801 |  |
| Age | -.870 | .605 | -1.437 | .154 |  |
| IL-6 | .154 | .272 | .566 | .573 |  |
| *T. gondii* x Age | 42.354 | 76.898 | .551 | .583 |  |
| *T. gondii* x IL-6 | -20.984 | 32.430 | -.647 | .519 |  |
| Age x IL-6 | -.470 | 1.193 | -.394 | .695 |  |
| *T. gondii* x Age x IL-6 *^b)^* | .257 | .515 | .500 | .618 |  |
| *N* = 107; *R* = .642; *R^2^* = .412; *F*(7, 99) = 9.92; *p* < .001; Δ*R²* = .001 *^b)^* | | | | | |
| Moderator: **IL-8** | | | | | |
| Intercept | 93.913 | 58.773 | 1.598 | .113 |  |
| *T. gondii* | -6.549 | 24.158 | -.271 | .787 |  |
| Age | -1.242 | 1.096 | -1.133 | .260 |  |
| IL-8 | .355 | .467 | .761 | .448 |  |
| *T. gondii* x Age | 29.204 | 75.816 | .385 | .701 |  |
| *T. gondii* x IL-8 | -14.692 | 32.327 | -.454 | .650 |  |
| Age x IL-8 | -.044 | 1.234 | -.035 | .972 |  |
| *T. gondii* x Age x IL-8 *^b)^* | .030 | .536 | .056 | .955 |  |
| *N* = 107; *R* = .632; *R^2^* = .399; *F*(7, 97) = 9.19; *p* < .001; Δ*R²* = .000 *^b)^* | | | | |  |
| Moderator: **IL-18** | | | | |  |
| Intercept | 39.554 | 176.699 | .224 | .823 |  |
| *T. gondii* | 45.813 | 76.693 | .597 | .552 |  |
| Age | -.554 | 2.576 | -.215 | .830 |  |
| IL-18 | -.373 | 1.124 | -.332 | .741 |  |
| *T. gondii* x Age | 19.124 | 97.036 | .197 | .844 |  |
| *T. gondii* x IL-18 | -24.421 | 42.096 | -.580 | .563 |  |
| Age x IL-18 | .067 | 1.442 | .047 | .963 |  |
| *T. gondii* x Age x IL-18 *^b)^*  *^b)^* | .206 | .630 | .327 | .744 |  |
| *N* = 100; *R* = .703; *R^2^* = .494; *F*(7, 92) = 12.81; *p* < .001, Δ*R²* = .001 *^b)^* | | | | |  |
| Moderator: **TNF-α** | | | | |  |
| Intercept | 79.790 | 83.697 | .953 | .343 |  |
| *T. gondii* | -32.863 | 37.451 | -.877 | .383 |  |
| Age | -.158 | 1.369 | -.115 | .909 |  |
| TNF-α | .623 | .619 | 1.007 | .317 |  |
| *T. gondii* x Age | -22.689 | 187.824 | -.121 | .904 |  |
| *T. gondii* x TNF-α | 19.428 | 79.223 | .245 | .807 |  |
| Age x TNF-α | .972 | 3.055 | .318 | .751 |  |
| *T. gondii* x Age x TNF-α *^b)^* | -.590 | 1.312 | -.450 | .654 |  |
| *N* = 103; *R* = .652; *R^2^* = .425; *F*(7, 95) = 10.02; *p* < .001, Δ*R²* = .005 *^b)^* | | | | |  |
| *^a)^ regression coefficients (b) with standard errors (SE), t- and p-values* | | | | |  |
| *^b)^ ΔR²: change in R^2^ due to the three-way interaction term* | | | | |  |

Table S12. Results of the moderator analyses with the LPS-3 as dependent variable, age as independent variable, and *T. gondii* and log10 transformed IL-6, IL-8, IL-18 and TNF-α as moderator variables. Δ*R²* indicates changes of the *R^2^* by the three-way interaction term.Bold text indicates significance.

| Outcome: **LPS-3** | *b ^a)^* | *SE ^a)^* | *t ^a)^* | *p ^a)^* |  |
| --- | --- | --- | --- | --- | --- |
| Moderator: **IL-6** | | | | | |
| Intercept | 47.854 | 24.122 | 1.984 | .053 |  |
| *T. gondii* | -4.304 | 10.124 | -.425 | .673 |  |
| Age | -.373 | .563 | -.662 | .511 |  |
| IL-6 | .063 | .236 | .266 | .792 |  |
| *T. gondii* x Age | -63.774 | 67.625 | -.943 | .351 |  |
| *T. gondii* x IL-6 | 24.116 | 28.175 | .856 | .397 |  |
| Age x IL-6 | 1.581 | 1.371 | 1.153 | .255 |  |
| *T. gondii* x Age x IL-6 *^b)^* | -.628 | .577 | -1.089 | .282 |  |
| *N* = 53; *R* = .510; *R^2^* = .260; *F*(7, 45) = 2.26; *p* < .05; Δ*R²* = .019 *^b)^* | | | | | |
| Moderator: **IL-8** | | | | | |
| Intercept | 57.661 | 54.373 | 1.060 | .295 |  |
| *T. gondii* | -7.073 | 21.925 | -.323 | .749 |  |
| Age | -.522 | 1.265 | -.413 | .682 |  |
| IL-8 | .103 | .517 | .199 | .843 |  |
| *T. gondii* x Age | -27.024 | 78.051 | -.346 | .731 |  |
| *T. gondii* x IL-8 | 7.831 | 32.511 | .241 | .811 |  |
| Age x IL-8 | .667 | 1.703 | .392 | .697 |  |
| *T. gondii* x Age x IL-8 *^b)^* | -.225 | .708 | -.317 | .753 |  |
| *N* = 51; *R* = .473; *R^2^* = .224; *F*(7, 43) = 1.77; *p* = .118; Δ*R²* = .002 *^b)^* | | | | |  |
| Moderator: **IL-18** | | | | |  |
| Intercept | -1.322 | 172.504 | -.008 | .994 |  |
| *T. gondii* | 16.850 | 73.396 | .230 | .819 |  |
| Age | -.756 | 3.800 | -.199 | .843 |  |
| IL-18 | .278 | 1.630 | .171 | .865 |  |
| *T. gondii* x Age | 16.756 | 93.282 | .180 | .858 |  |
| *T. gondii* x IL-18 | -7.767 | 39.609 | -.196 | .845 |  |
| Age x IL-18 | .453 | 2.045 | .221 | .826 |  |
| *T. gondii* x Age x IL-18 *^b)^*  *^b)^* | -.213 | .876 | -.243 | .809 |  |
| *N* = 53; *R* = .499; *R^2^* = .249; *F*(7, 92) = 2.13; *p* = .059, Δ*R²* = .001 *^b)^* | | | | |  |
| Moderator: **TNF-α** | | | | |  |
| Intercept | 30.075 | 22.775 | 1.321 | .194 |  |
| *T. gondii* | 2.446 | 10.052 | .243 | .809 |  |
| Age | .213 | .508 | .420 | .677 |  |
| TNF-α | -.172 | .223 | -.773 | .444 |  |
| *T. gondii* x Age | 2.828 | 52.577 | .054 | .957 |  |
| *T. gondii* x TNF-α | -2.344 | 21.999 | -.107 | .916 |  |
| Age x TNF-α | .488 | 1.255 | .389 | .700 |  |
| *T. gondii* x Age x TNF-α *^b)^* | -.183 | .525 | -.349 | .729 |  |
| *N* = 49; *R* = .573; *R^2^* = .328; *F*(7, 41) < 1; *p* = .729, Δ*R²* = .002 *^b)^* | | | | |  |
| *^a)^ regression coefficients (b) with standard errors (SE), t- and p-values* | | | | |  |
| *^b)^ ΔR²: change in R^2^ due to the three-way interaction term* | | | | |  |

Table S13. Results of the moderator analyses with the LPS-7 as dependent variable, age as independent variable, and *T. gondii* and log10 transformed IL-6, IL-8, IL-18 and TNF-α as moderator variables. Δ*R²* indicates changes of the *R^2^* by the three-way interaction term. Bold text indicates significance.

| Outcome: **LPS-7** | *b ^a)^* | *SE ^a)^* | *t ^a)^* | *p ^a)^* |  |
| --- | --- | --- | --- | --- | --- |
| Moderator: **IL-6** | | | | | |
| Intercept | 27.432 | 35.233 | .779 | .440 |  |
| *T. gondii* | 1.750 | 14.787 | .118 | .906 |  |
| Age | -.293 | .823 | -.357 | .723 |  |
| IL-6 | .036 | .345 | .104 | .918 |  |
| *T. gondii* x Age | -49.393 | 98.774 | -.500 | .619 |  |
| *T. gondii* x IL-6 | 19.958 | 41.152 | .485 | .630 |  |
| Age x IL-6 | 1.564 | 2.002 | .781 | .439 |  |
| *T. gondii* x Age x IL-6 *^b)^* | -.649 | .842 | -.770 | .445 |  |
| *N* = 51; *R* = .344; *R^2^* = .118; *F*(7, 45) < 1; *p* = .543; Δ*R²* = .012 *^b)^* | | | | | |
| Moderator: **IL-8** | | | | | |
| Intercept | 51.972 | 79.377 | .655 | .516 |  |
| *T. gondii* | -6.217 | 32.007 | -.194 | .847 |  |
| Age | -.464 | 1.847 | -.251 | .803 |  |
| IL-8 | .074 | .755 | .098 | .923 |  |
| *T. gondii* x Age | -55.585 | 113.944 | -.488 | .628 |  |
| *T. gondii* x IL-8 | 19.883 | 47.463 | .419 | .677 |  |
| Age x IL-8 | .964 | 2.486 | .388 | .700 |  |
| *T. gondii* x Age x IL-8 *^b)^* | -.354 | 1.034 | -.343 | .733 |  |
| *N* = 51; *R* = .325; *R^2^* = .106; *F*(7, 45) < 1; *p* = .651; Δ*R²* = .019 *^b)^* | | | | |  |
| Moderator: **IL-18** | | | | |  |
| Intercept | 167.648 | 237.371 | .706 | .484 |  |
| *T. gondii* | -40.843 | 100.995 | -.404 | .688 |  |
| Age | -6.032 | 5.229 | -1.154 | .255 |  |
| IL-18 | 2.114 | 2.243 | .943 | .351 |  |
| *T. gondii* x Age | -86.117 | 128.359 | -.671 | .506 |  |
| *T. gondii* x IL-18 | 27.202 | 54.504 | .499 | .620 |  |
| Age x IL-18 | 3.390 | 2.814 | 1.205 | .235 |  |
| *T. gondii* x Age x IL-18 *^b)^*  *^b)^* | -1.242 | 1.205 | -1.030 | .308 |  |
| *N* = 53; *R* = .454; *R^2^* = .206; *F*(7, 45) = 1.67; *p* = .141, Δ*R²* = .019 *^b)^* | | | | |  |
| Moderator: **TNF-α** | | | | |  |
| Intercept | 11.763 | 37.609 | .313 | .756 |  |
| *T. gondii* | 8.008 | 16.599 | .482 | .632 |  |
| Age | .251 | .839 | .299 | .767 |  |
| TNF-α | -.185 | .368 | -.503 | .618 |  |
| *T. gondii* x Age | -7.371 | 86.822 | -.085 | .933 |  |
| *T. gondii* x TNF-α | .248 | 36.328 | .007 | .995 |  |
| Age x TNF-α | .427 | 2.072 | .206 | .838 |  |
| *T. gondii* x Age x TNF-α *^b)^* | -.122 | .866 | -.141 | .889 |  |
| *N* = 49; *R* = .358; *R^2^* = .128; *F*(7, 41) < 1; *p* = .544 Δ*R²* = .000 *^b)^* | | | | |  |
| *^a)^ regression coefficients (b) with standard errors (SE), t- and p-values* | | | | |  |
| *^b)^ ΔR²: change in R^2^ due to the three-way interaction term* | | | | |  |
